# Diversity and Pathogenicity of *Botryosphaeriaceae* Species Isolated from Olives in Istria, Croatia, and Evaluation of Varietal Resistance

**DOI:** 10.3390/plants13131813

**Published:** 2024-07-01

**Authors:** Elena Petrović, Karolina Vrandečić, Andreina Belušić Vozila, Jasenka Ćosić, Sara Godena

**Affiliations:** 1 Institute of Agriculture and Tourism, Karla Huguesa 8, 52440 Poreč, Croatia; andreina@iptpo.hr (A.B.V.); sara@iptpo.hr (S.G.); 2 Faculty of Agrobiotechnical Sciences Osijek, Josip Juraj Strossmayer University of Osijek, Vladimira Preloga 1, 31000 Osijek, Croatia; kvrandecic@fazos.hr (K.V.); jcosic@fazos.hr (J.Ć.)

**Keywords:** Botryosphaeria dieback, *Botryosphaeria dothidea*, *Diplodia* spp., *Dothiorella* spp., first report, *Neofusicoccum* sp.

## Abstract

During 2021 and 2022, a field investigation was conducted in Istria, Croatia, searching for trees exhibiting signs of Botryosphaeria dieback. Samples of symptomatic trees were collected from 26 different locations and analysed. Isolates that morphologically corresponded to species from the *Botryosphaeriaceae* family were selected, and detailed morphological characterisation and molecular identification of the isolates were conducted. Based on morphological characteristics and phylogenetic analysis using the internal transcribed spacer (ITS), beta-tubulin (*TUB2*), and translation elongation factor 1-alpha (*TEF1-α*) regions, six species of fungi from the *Botryosphaeriaceae* family were identified: *Botryosphaeria dothidea* (Moug. ex Fr.) Ces. & De Not.; *Diplodia mutila* (Fr.) Fr.; *Diplodia seriata* De Not.; *Dothiorella iberica* A.J.L. Phillips, J. Luque & A. Alves; *Dothiorella sarmentorum* (Fr.) A.J.L. Phillips, Alves & Luque; and *Neofusicoccum parvum* (Pennycook & Samuels) Crous, Slippers & A.J.L. Phillips. This is the first report of *D. mutila*, *Do. sarmentorum*, and *Do. iberica* causing Botryosphaeria dieback on olive trees in Croatia, and the first study investigating the resistance of Croatian olive varieties to species from the *Botryosphaeriaceae* family. Pathogenicity testing of selected isolates and assessment of variety resistance were conducted on four different olive varieties, namely Buža, Istarska bjelica, Leccino, and Rosinjola, using representative isolates of the mentioned species. The most aggressive species was found to be *N. parvum*. Olive varieties exhibited differences in susceptibility depending on the fungus they were infected with.

## 1. Introduction

The olive (*Olea europea* L.) is one of the oldest cultivated plants, spread across the Mediterranean from 45° north to 35° south [1]. Material evidence from ancient times indicates the significance of olive growing and olive oil, mostly used as food, fuel for oil lamps, cosmetic purposes, etc. [1]. Numerous tales suggest how olive production expanded. The prevalence of olives in Istria, as well as in Sicily and southern Spain, is attributed by historians to Aristeus, the ancient pastoral god of the Arcadians, Boeotians, and Thessalians, who was considered the inventor of olives and olive oil [2,3]. Pribetić [1] suggests the assumption that the olive’s homeland is Palestine or Asia Minor, from where it first spread to Egypt. Mythology tells of the goddess Minerva, challenged to a contest by Neptune, who plucked the first olive plant from the ground, already in bloom and bearing fruit, thus making it a symbol of peace [4]. It is interesting to note that a golden olive branch was left on the moon’s surface by Apollo 11 crew members as a symbol of peace. Olive cultivation in Istria dates back to the 1st century BC, mainly along the western and southern coastal areas [5]. During the period between 1500 and 1700, there was a significant decline in production, but in the 19th and 20th centuries, its production once again spread throughout the Istrian Peninsula [3]. As stated by Godena et al. [6], the Istrian Peninsula stands as the northernmost olive-growing region in Croatia and is also one of the northernmost olive-growing regions globally. Croatia’s olive strategy lies in producing extra-high-quality olive oil, precisely due to suitable climatic conditions [7]. According to the FAO [8], there are no data available for the annual olive production in the world for the year 2023, but in 2022, the production amounted to 21.4 million t. Olive production in Croatia amounted to approximately 40,100 t in 2022 [8], and 29,800 t in 2023 [9]. The production of a high-quality olive oil strongly depends on the quality of the fruit from which the oil is extracted. However, the quality of the fruit depends on numerous factors, such as agroecological conditions, disease and pest attacks, varieties, etc.

Olive varieties exhibit a vast range of diversity. A key question is whether this differentiation occurred post-domestication or whether olives have multiple origins [10]. Typically, olives are propagated through cuttings or grafts, resulting in varieties that are essentially clones [10]. It is assumed that over 1000 varieties and types of olives are cultivated in the Mediterranean region [1]. According to Rugini [11], the number of cultivated olive varieties is estimated to be around 2500. In studies investigating the pathogenicity of fungi and the susceptibility of olive varieties, two terms frequently appear: olive variety and olive cultivar. When discussing olives and olive trees, there is no difference between these terms, as the term “cultivar” is short for “cultivated variety” and is used frequently in olive growing to refer to the different varieties of olives produced. Therefore, the olive cultivar is a synonym for olive variety [12,13]. A significant advantage of Croatian olive cultivation is the indigenous assortment that distinguishes certain areas with the uniqueness of olive oil aroma and flavour, especially since nowadays, oil and olives with a geographical origin achieve twice the price of oil without an origin [7]. In Istrian olive groves, slightly more than a third of the trees belong to indigenous varieties: Buža (50.69%), Istarska bjelica (30.22%), Rosinjola (5.72%), Crnica (syn. Karbonera, 5.60%), and other less represented varieties (7.77%). New plantations primarily consist of foreign varieties (Leccino, Frantoio, Pendolino) [5].

The Buža variety (syn. Buga, Burgaca, Domaća, Gura, Morgaca) [14] is widespread in Istria. Pribetić [1] lists it as the most widespread variety in that area. It is highly valued inland for its excellent oil. It is sensitive to early autumn cold, which reduces oil yields [3]. It is known that in Istria entire plantations suffered from low temperatures in certain years [1]. The name “Buža” comes from the ancient term “bugio,” meaning pitted, hollow. Hugues [3] suggests that the name of this variety could originate from the frequent cavities or holes in its trunk near the stump. In the Vodnjan area (Istria), the Buža variety is often grown. This variety is susceptible to peacock spot disease caused by the species *Venturia oleaginea* (Castagne) Rossman & Crous and the appearance of sooty mould, and it is susceptible to olive fruit fly (*Bactrocera oleae* Rossi) and olive moth (*Prays oleae* Bernard) [1].

The Rosinjola variety (syn. Rošinjola, Rosulja, Rovinječka, Rušinjola) [14] is mostly grown in the southern part of Istria, around Vrsar, Rovinj, and Vodnjan [1]. It produces good-quality oil, with an intense aroma and a pronounced bitter taste [1]. Due to its dense canopy, it is often attacked by insects, which causes the appearance of sooty mould, but it shows good resistance to other pests and diseases [1].

The Istarska bjelica variety (syn. Bjelica, Bjankera, Bianchera) [14] is also widespread in Istria and on the Kvarner islands. Unlike Buža, it is somewhat more resistant to low temperatures and the wind [1]. It has a high oil yield, abundant productivity, and excellent oil quality, but it is susceptible to *B. oleae* [1].

The Leccino variety (syn. Leccio) [14] originates from Italy, and it has been cultivated in Istria since 1940 [1]. It is one of the most widespread varieties globally due to its significant adaptability to different agroecological conditions [1]. The Leccino variety is resistant to low temperatures, suitable for intensive plantations, shows good and constant productivity and, finally, produces outstanding-quality oil [1]. Moreover, it is noted to be more resistant to peacock spot disease [1,7], olive knot disease caused by the bacteria *Pseudomonas savastanoi* pv. *savastanoi*, and the pest *P. oleae*, but it is susceptible to *B. oleae* [1].

A large number of fungi and pests have been reported to cause damage to olive trees. Among the most significant pest of olive trees is *B. oleae*, which is the most economically significant pest in Croatian olive cultivation. On the other hand, in terms of diseases, there is peacock spot disease, as well as patula, caused by *Botryosphaeria dothidea* (Moug. ex Fr.) Ces. & De Not., and anthracnose of fruits caused by the species *Colletotrichum gloeosporioides* (Penz.) Penz. & Sacc. (syn. *Gloeosporium olivarum*) [7]. Fungi from the *Botryosphaeriaceae* family identified as pathogens of olive trees in Croatia include the species *B. dothidea*, *Diplodia seriata* De Not. and *Neofusicoccum parvum* (Pennycook & Samuels) Crous, Slippers & A.J.L. Phillips [15,16,17,18]. In addition to the fungi from the *Botryosphaeriaceae* family, other pathogens of olive trees in Croatia include the species *Armillaria mellea* (Vahl) P. Kumm. [17]; *Biscogniauxia mediterranea* and *Biscogniauxia nummularia* (Bull.) Kuntze [19]; *Colletotrichum* spp. [15]; *Comoclathris incompta* (Sacc. & Martelli) Ariyaw. & K.D. Hyde (syn. *Phoma incompta* Sacc. & Mart.) [20]; *Cytospora pruinosa* Défago [21]; *Diaporthe* sp. [18]; *Nigrospora gorlenkoana* Novobr., *Nigrospora osmanthi* Mei Wang & L. Cai, and *Nigrospora philosophiae-doctoris* M. Raza, Qian Chen & L. Cai [22]; *Phaeoacremonium iranianum* L. Mostert, Gräfenhan, W. Gams & Crous [23]; *Pleurostoma richardsiae* (Nannfeldt) Réblová & Jaklitsch [24]; *Pseudocercospora cladosporioides* (Sacc.) U. Braun [15]; *Sordaria fimicola* (Roberge ex Desm.) Ces. & De Not [19]; *Venturia oleaginea* (Castagne) Rossman & Crous (syn. *Spilocaea oleaginea*) [25,26]; and *Verticillium dahliae* Klebahn [27].

Peacock spot disease and patula are among the first described fungal diseases of olives in Croatia. Peacock spot disease was first described in 1901 in Croatia [28]. Patula (syn. Dalmatian disease, Botryosphaeria dieback, escudete, maricume delle drupe, lepre des olives, etc.) was first described in 1883 in Dalmatia by the German–Australian botanist Thumen, and now it is present in almost all olive-growing countries in the Mediterranean region [7]. It compromises the quality of oil and diminishes the value of table olives. The vector of *B. dothidea* is the olive fruit fly and its predator *Lasioptera berlesiana* Paoli (syn. *Prolasioptera berlesiana*). *L. berlesiana* carries *B. dothidea* spores in a mycangium. While the mosquito deposits its egg adjacent to the fly egg, it also inoculates the puncture made by *B. olea* with the fungus [29]. The symptoms of the disease resemble an attack by *C. gloeosporioides*. Signs of the disease in commercial groves encompass necrotic, sunken, and distinctly demarcated lesions on fruits [30]. Besides *B. dothidea*, fungi from the *Botryosphaeriaceae* family are known as some of the most common pathogens of olive trees [31,32]. For instance, causal agents of Botryosphaeria dieback and fruit rot include species such as *D. olivarum* A.J.L. Phillips & Lazzizera; *N. vitifusiforme* (Van Niekerk & Crous) Crous, Slippers & A.J.L. Phillips; *N. parvum*; and *N. mediterraneum* Crous, M.J. Wingf. & A.J.L. Phillips [33,34]. Disease symptoms can be observed on olive fruits, leaves, branches, and trunks. They cause fruit rot, leaf wilting and defoliation, bark discolouration, branch dieback, canker formations, and the appearance of necrosis [35,36]. Fruit contamination with fungi is also a concern due to mycotoxins, which can develop through fruit decay and prolonged storage of damaged fruit [7].

The *Botryosphaeriaceae* family belongs to the class *Dothideomycetes* and the order *Botryosphaeriales*. It encompasses a range of morphologically diverse fungi that can be pathogens, endophytes, or saprobes, primarily on woody hosts [37]. This family was introduced by Theissen & Sydow in 1918 as a sub-family of *Pseudosphaeriaceae*. These species spread through conidia [38], with conidiomata (pycnidia), the fungi’s fruiting bodies, releasing conidia during rain and high humidity [39]. Fungi from the *Botryosphaeriaceae* family are also found in healthy tissue parts of the plant and usually cause diseases after the plant is exposed to stressful conditions (post-harvest and heavy rain). These species can remain in a latent stage until favourable conditions for development arise (biotic and/or abiotic stress) [40]. Species from this family are considered aggressive pathogens. According to Schoch et al. [41], 33 genera with over 1200 species of fungi in this family have been identified. The MycoBank database currently encompass 85 genera of fungi in this family [42].

The overall objectives of this study were to identify the causative agent responsible for symptoms observed in olive trees in Istria, Croatia; to conduct morphological characterisation and molecular identification of the fungal isolates through PCR and DNA sequencing of the ITS, *TUB2*, and *TEF1-α* gene regions; to evaluate the pathogenicity of fungal isolates via pathogenicity tests; and to investigate the resistance of various olive varieties to the identified fungal species.

## 2. Results

### 2.1. Field Symptoms

**Figure 1:**
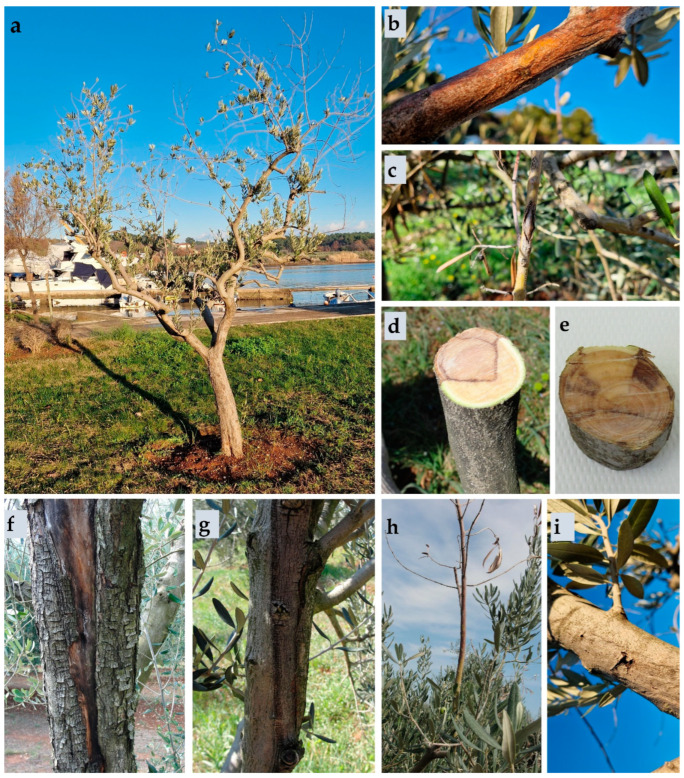
Symptoms of infection observed during field research caused by fungi from the *Botryosphaeriaceae* family: (**a**) branch and twig dieback and defoliation caused by the species *Botriosphaeria dothidea*; (**b**) reddish-yellow discolouration of bark caused by the species *B. dothidea*; (**c**) cracking of twig bark, branch dieback, and leaf wilting caused by the species *Diplodia mutila*; (**d**,**e**) necrotic changes in branch cross-sections: (**d**) *Neofusicoccum parvum*, (**e**) *Diplodia seriata*; (**f**,**g**) cracking of tree bark and the appearance of necrotic lesions: (**f**) *Dothiorella sarmentorum*, (**g**) *N. parvum*; (**h**) branch drying and bark discolouration caused by the species *Dothiorella iberica*; (**i**) bark cracking and the appearance of necrosis caused by the species *B. dothidea*.

Symptoms observed during field research included branch and twig dieback, leaf wilting and defoliation, fruit rot, bark cracking, reddish-brown discolouration of bark, and the appearance of dark-brown necrotic lesions. Necrotic lesions were particularly evident in cross-sectional branch cuts (Figure 1). 

Out of the 26 locations visited in total, species from the *Botryosphaeriaceae* family were identified in 10, representing 38.46% of the total. Among the 112 sampled trees, species from the *Botryosphaeriaceae* family were identified in 13, making up 11.6% of the total count. The species *B. dothidea* and *N. parvum* were found at four locations in Istria, while the species *Do. sarmentorum* was found at two locations. The species *D. mutila*, *D. seriata*, and *Do. iberica* were each found at one location. The highest number of isolates was collected in Vodnjan (5), followed by Rovinj (4), Poreč (3), and Novigrad (1). 

None of the olive groves were equipped with irrigation systems. Pruning was carried out manually at every location, except for grove R18, where a combination of manual and mechanical methods was utilised. Moreover, pruning and burning of plant residues were standard practices across these olive groves. More information about the agricultural practices implemented in olive groves is presented in Table 1. 

### 2.2. Morphological Characterisation

#### 2.2.1. Botryosphaeria Dothidea

**Figure 2:**
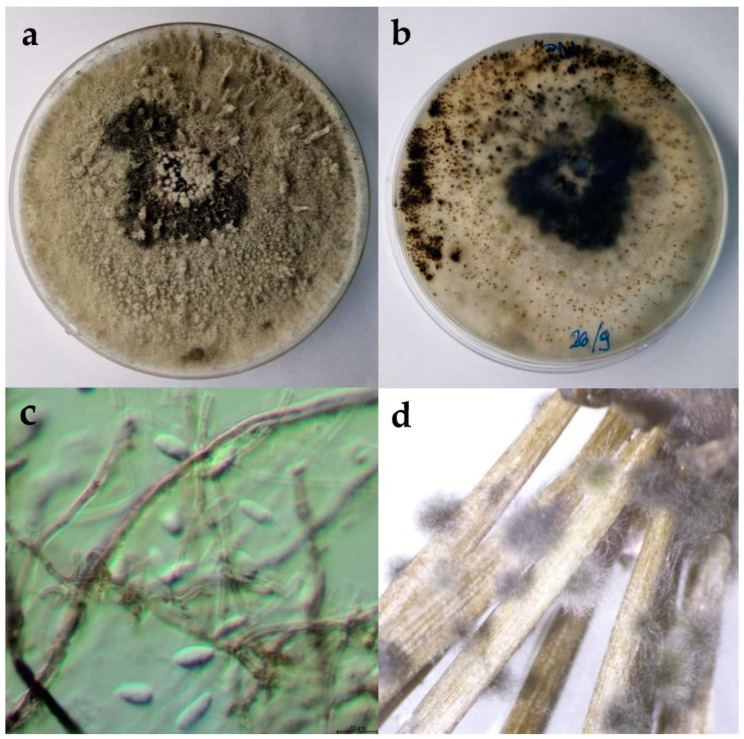
(**a**,**b**) Upper and reverse view of *Botryospheria dothidea* isolate 15 days after incubation at 25 °C on potato dextrose agar (PDA) medium; (**c**) hyphae and conidia of *B. dothidea* isolate observed under the microscope, scale bar = 20 μm; (**d**) pycnidia developed on WA + *Pinus.*

The colonies expanded to a diameter of nine cm within five days at 25 °C on potato dextrose agar (PDA) and after seven days on water agar (WA). On WA, the mycelium was poorly developed, ranging in colour from white to greyish. On PDA, the colony colour in the initial stages of growth was white-grey, gradually turning olive green to grey with white tips. As the colony aged, it developed a black-grey colour. The mycelium was dense and matte, with a woolly, cottony, and fluffy appearance (Figure 2). The reverse colonies were white to olivaceous green with dark grey spots. The hyphae were septate, branched, and hyaline. Pycnidia formed on the nutrient medium WA + *Pinus*. The pycnidia were fluffy, olivaceous green with white tips, appearing individually or in clusters. Conidia were fusiform, thicker in the middle and thinner at the ends, aseptate, and hyaline to brownish. The dimensions of the conidia are shown in Table 2.

#### 2.2.2. Diplodia Mutila

**Figure 3:**
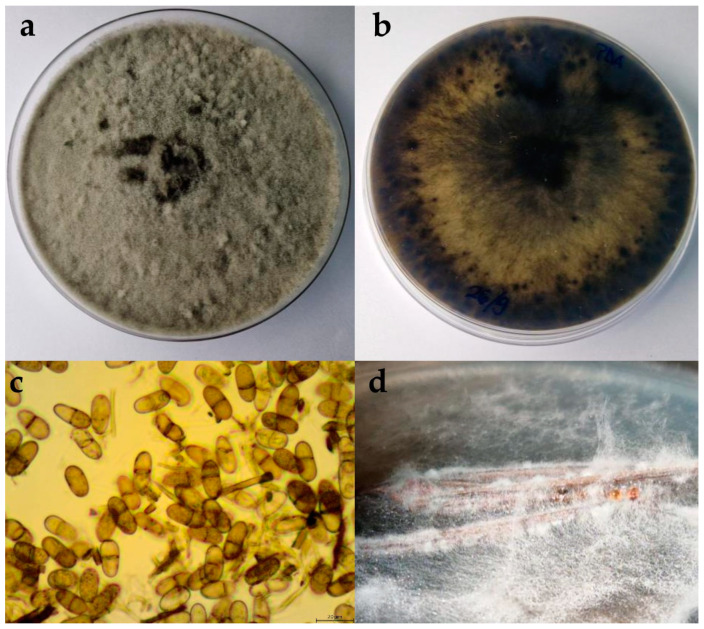
(**a**,**b**) Upper and reverse view of *Diplodia mutila* isolate 15 days after incubation at 25 °C on potato dextrose agar (PDA) medium; (**c**) conidia of *D. mutila* isolate observed under the microscope, scale bar = 20 μm; (**d**) pycnidia developed on WA + *Pinus.*

The colonies expanded to a diameter of nine cm within four days at 25 °C on PDA and after seven days on WA. On WA, the mycelium was poorly developed, ranging in colour from white to greyish. On PDA, the colony colour was olivaceous green. As the colony aged, it developed a black-grey colour. The mycelium was dense and matte, with a woolly, cottony, and fluffy appearance (Figure 3). The reverse colonies were white with black spots. The hyphae were septate, branched, and hyaline. Pycnidia formed on the nutrient medium WA + *Pinus*. The pycnidia were fluffy and white, appearing individually or in clusters. Conidia were capsule shaped, hyaline, and aseptate, and over time changed from beige to dark brown, with one septum. The dimensions of the conidia are shown in Table 2.

#### 2.2.3. Diplodia Seriata

**Figure 4:**
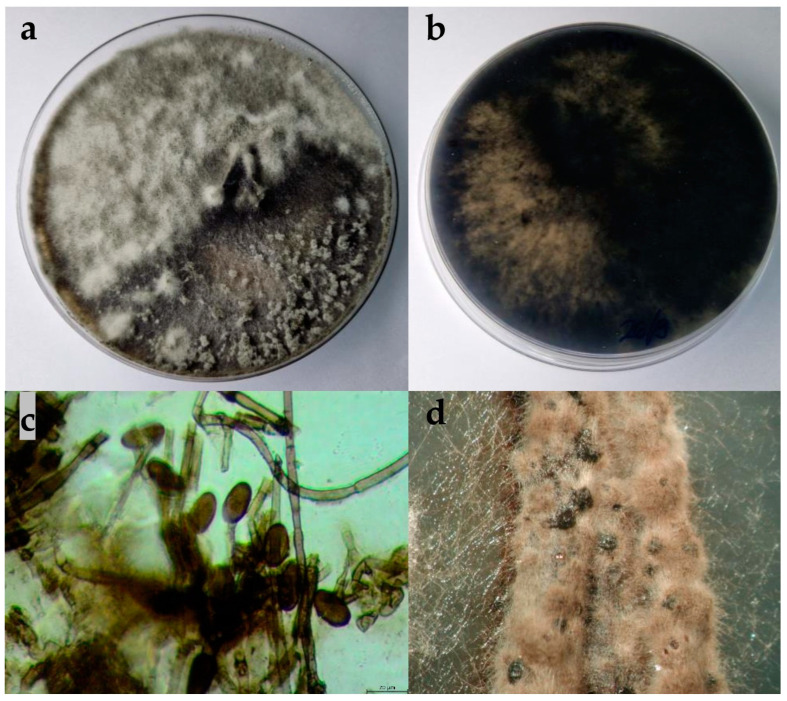
(**a**,**b**) Upper and reverse view of *Diplodia seriata* isolate 15 days after incubation at 25 °C on potato dextrose agar (PDA) medium; (**c**) hyphae and conidia of *D. seriata* isolate observed under the microscope, scale bar = 20 μm; (**d**) pycnidia developed on WA + *Pinus.*

The colonies expanded to a diameter of nine cm within four days at 25 °C on PDA and after seven days on WA. On WA, the mycelium was poorly developed, ranging in colour from white to greyish. On PDA, the colony colour was white-grey. As the colony aged, it developed a dark grey colour. The mycelium was dense and matte, with a woolly, cottony, and fluffy appearance (Figure 4). In certain areas, fluffy, whitish clumps of mycelium formed. The reverse colonies were dark grey. The hyphae were septate, branched, and hyaline. Pycnidia formed on the nutrient medium WA + *Pinus*. The pycnidia were fluffy and brown with white tips, appearing in clusters. The conidia were ovoid, rounded or slightly pointed at the edges, aseptate, and hyaline, and over time turned dark brown. The dimensions of the conidia are shown in Table 2.

#### 2.2.4. Dothiorella Iberica

**Figure 5:**
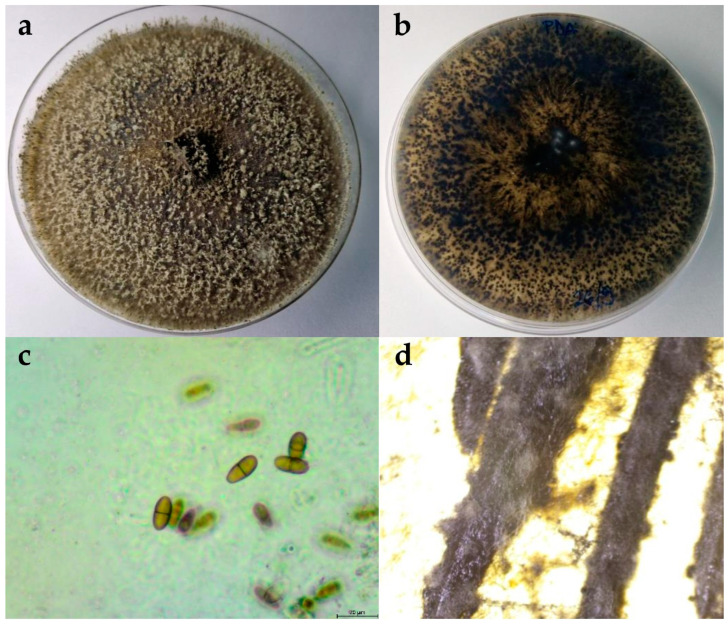
(**a**,**b**) Upper and reverse view of *Dothiorella iberica* isolate 15 days after incubation at 25 °C on potato dextrose agar (PDA) medium; (**c**) conidia of *D. iberica* isolate observed under the microscope, scale bar = 20 μm; (**d**) pycnidia developed on WA + *Pinus.*

The colonies expanded to a diameter of nine cm within four days at 25 °C on PDA and after eight days on WA. On WA, the mycelium was poorly developed, ranging in colour from white to greyish. On PDA, the colony colour was dark grey. The mycelium was dense and matte, with a cottony appearance (Figure 5). The aerial mycelium was more adherent to the substrate, forming rounded cushions, and was not as fluffy as in the previously mentioned species. The reverse colonies were dark grey. The hyphae were septate, branched, and hyaline. Pycnidia formed on the nutrient medium WA + *Pinus*. The pycnidia were fluffy and grey, appearing in clusters. Conidia were capsule shaped, rounded at the edges, and hyaline, and over time turned light brown, with one septum. The dimensions of the conidia are shown in Table 2.

#### 2.2.5. Dothiorella Sarmentorum

**Figure 6:**
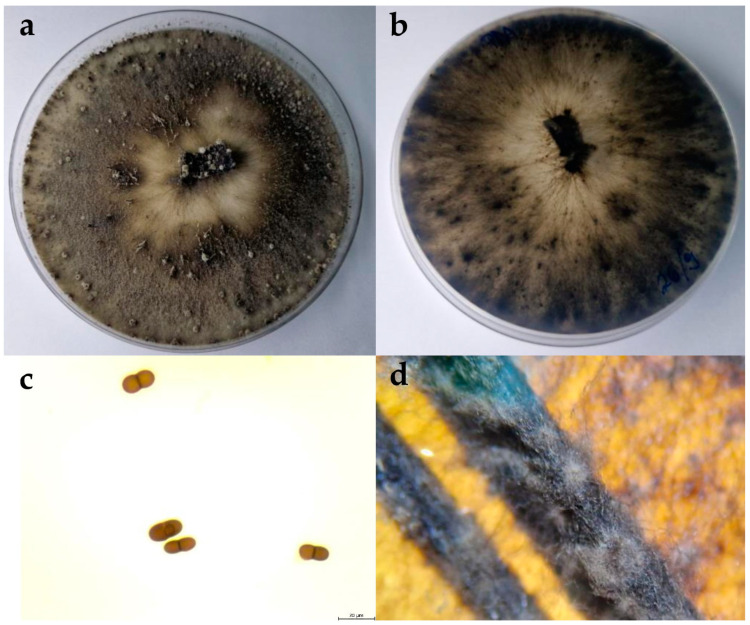
(**a**,**b**) Upper and reverse view of *Dothiorella sarmentorum* isolate 15 days after incubation at 25 °C on potato dextrose agar (PDA) medium; (**c**) conidia of *D. sarmentorum* isolate observed under the microscope, scale bar = 20 μm; (**d**) pycnidia developed on WA + *Pinus*.

The colonies expanded to a diameter of nine cm within three days at 25 °C on PDA and after eight days on WA. On WA, the mycelium was poorly developed, ranging in colour from white to greyish. On PDA, the colony colour was dark grey with a white coating and brown edges. The mycelium was dense and matte, with a cottony appearance (Figure 6). The mycelium was adherent to the substrate with little cushion-like protrusions. The reverse colonies were black with brown edges. The hyphae were septate, branched, and hyaline. Pycnidia formed on the nutrient medium WA + *Pinus*. The pycnidia were fluffy and brown with white tips, appearing in clusters. The conidia were capsule-shaped, rounded at the edges, hyaline, and aseptate, and over time turned light brown, with one septum. The dimensions of the conidia are shown in Table 2.

#### 2.2.6. Neofusicoccum Parvum

**Figure 7:**
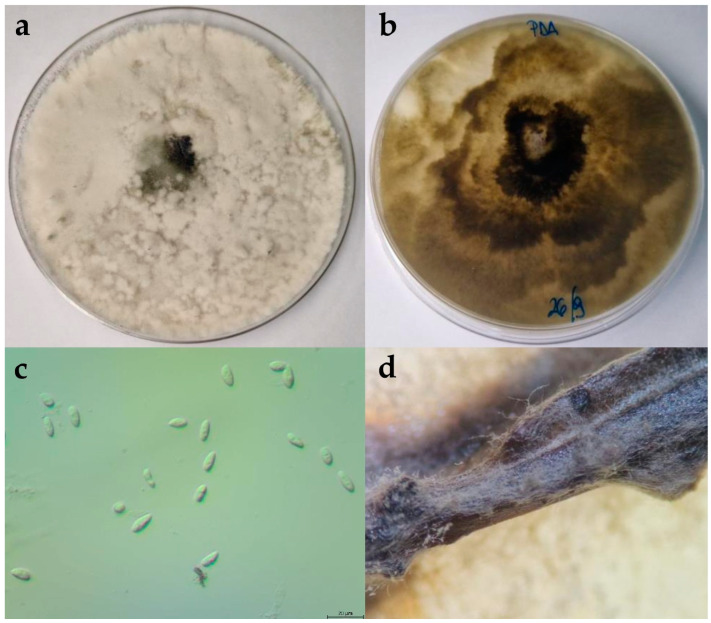
(**a**,**b**) Upper and reverse view of *Neofusicoccum parvum* isolate 15 days after incubation at 25 °C on potato dextrose agar (PDA) medium; (**c**) conidia of *N. parvum* isolate observed under the microscope, scale bar = 20 μm; (**d**) pycnidia developed on WA + *Pinus.*

The colonies expanded to a diameter of nine cm within four days at 25 °C on PDA and after six days on WA. On WA, the mycelium was poorly developed, ranging in colour from white to greyish. On PDA, the colony colour varied among isolates, ranging from light grey to brownish grey, and up to dark grey. The mycelium was dense and matte, with a cottony appearance (Figure 7). The aerial mycelium was adherent to the substrate. The reverse colonies were dark grey with beige-brown edges. The hyphae were septate, branched, and hyaline. Pycnidia formed on the nutrient medium WA + *Pinus*. The pycnidia were fluffy and grey with white tips, appearing in clusters. The conidia were ellipsoidal, with a round apex but sometimes slightly pointed at the ends, aseptate, and hyaline. The dimensions of the conidia are shown in Table 2.

#### 2.2.7. Rate of Mycelial Growth

In tests of mycelial growth rate conducted at eight different temperatures, after 48 h, none of the isolates showed growth at 5 °C or 40 °C. At 10 °C, 15 °C, 20 °C, and 25 °C, the species *Do. sarmentorum*, *Do. iberica*, and *D. seriata* exhibited the fastest growth rates. At 25 °C, the highest growth was recorded for all isolates except for the species *N. parvum*, which had the highest growth at 30 °C (Table 3). According to empirically derived data, a temperature of 25 °C proved optimal for the growth of *B. dothidea*, *D. seriata*, *D. mutila*, *Do. iberica*, and *Do. sarmentorum*, while for *N. parvum*, the optimal temperature was 30 °C. At 30 °C, the growth rate of *Do. iberica* and *Do. sarmentorum* drastically decreased. At 35 °C, *B. dothidea* exhibited the fastest growth, followed by *N. parvum*, *D. seriata*, and *D. mutila*, whereas *Do. iberica* and *Do. sarmentorum* did not show any mycelial growth. 

According to the empirical mathematical modelling (Table 4), optimal temperatures for species growth ranged between 21.3 °C and 28.1 °C, minimum temperatures between 5.1 °C and 5.4 °C, and maximum temperatures between 31.8 °C and 39.9 °C. As evident from the obtained data, the highest optimal growth temperature was recorded for the species *N. parvum*, while the lowest optimal growth temperature was recorded for the species *Do. sarmentorum*.

### 2.3. Molecular Phylogenetic Identification

Six fungal species were identified as belonging to the *Botryosphaeriaceae* family. All 39 nucleotide sequences obtained from 13 isolates, analysed via BLAST in this study, exhibited a 100% match for the ITS, *TUB2*, and *TEF1-α* gene regions, closely aligning with the species listed in the GenBank database. The sequences generated from this research were added to the GenBank database and are available under the accession numbers indicated in Table 5.

**Figure 8:**
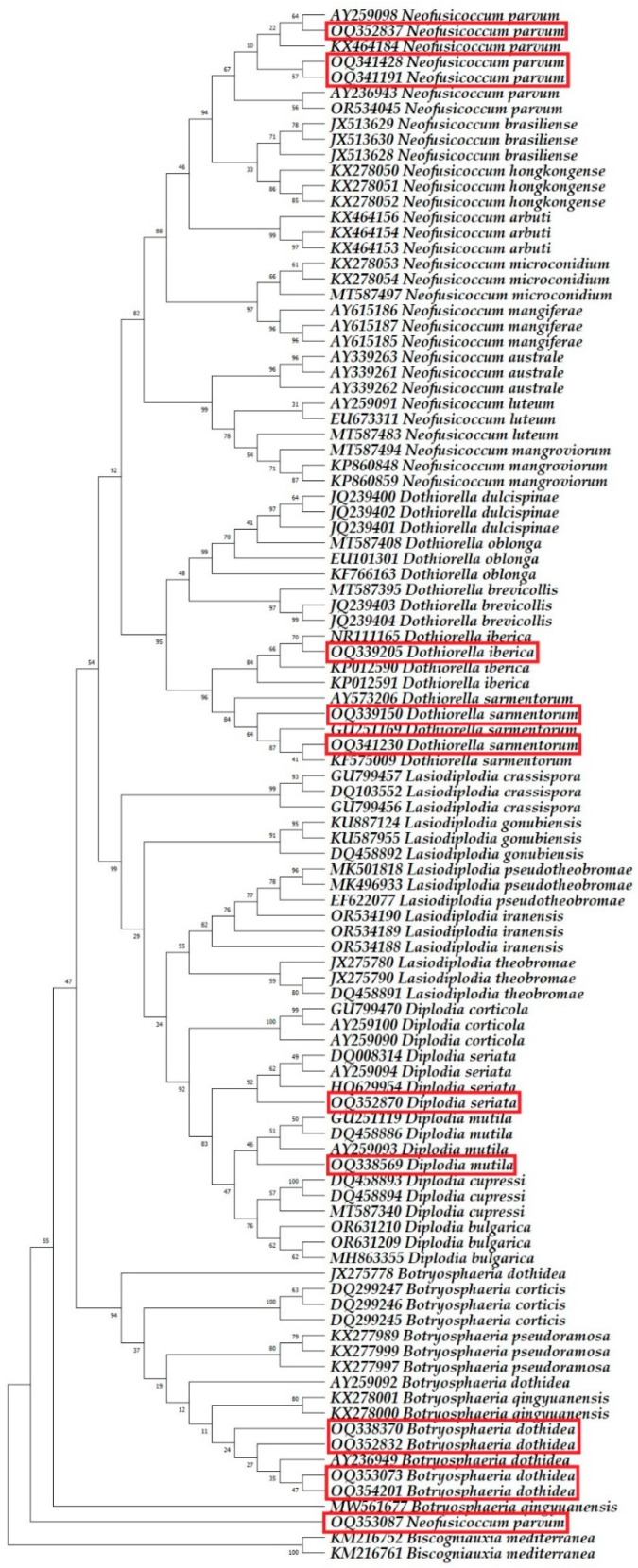
The phylogenetic tree, based on the alignment of internal transcribed spacer sequences, highlights the sequences identified in this research with red rectangles.

**Figure 9:**
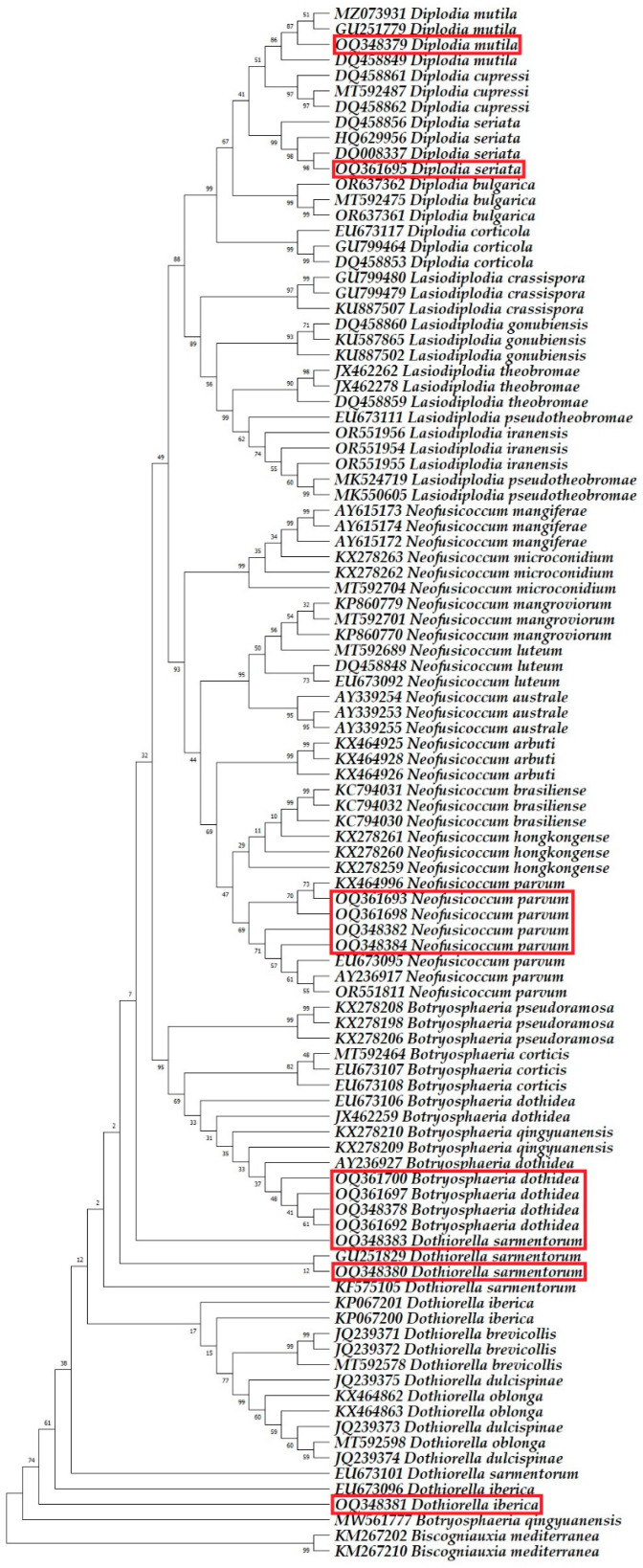
The phylogenetic tree, based on the alignment of beta-tubulin sequences, highlights the sequences identified in this research with red rectangles.

**Figure 10:**
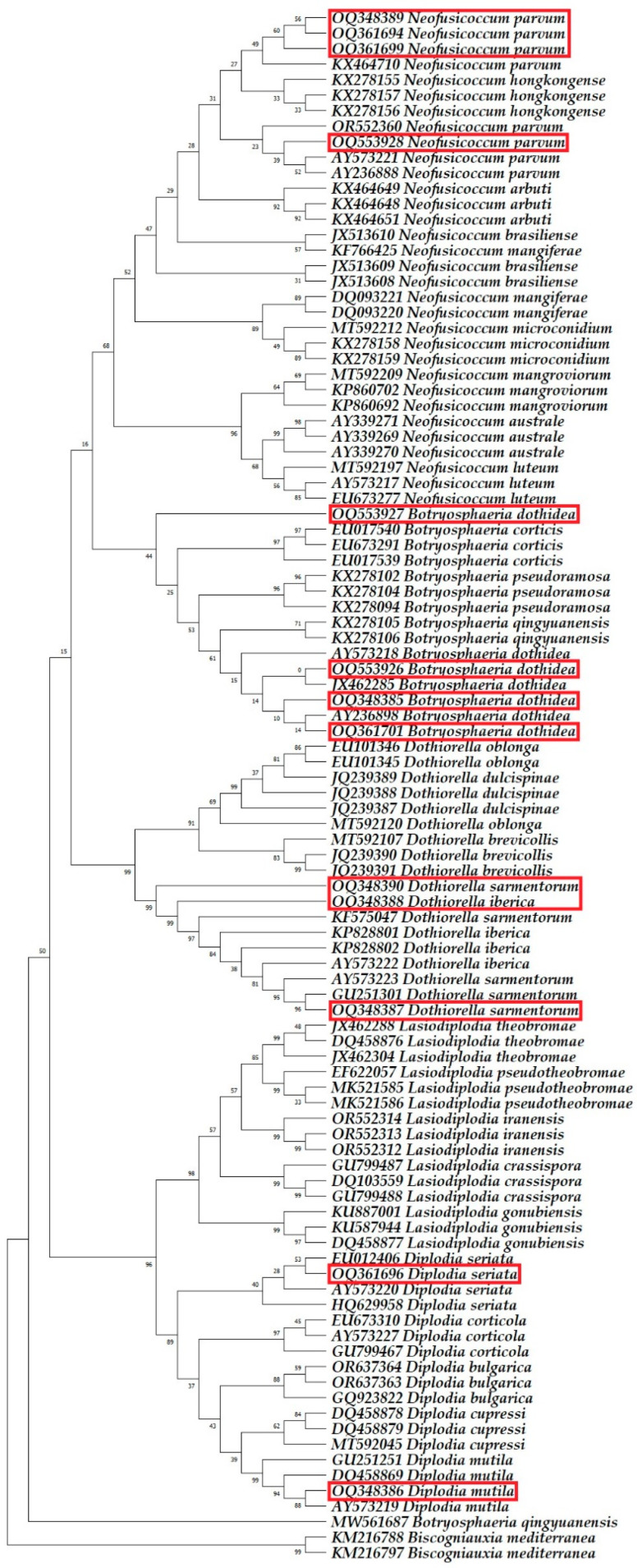
The phylogenetic tree, based on the alignment of translation elongation factor 1-alpha sequences, highlights the sequences identified in this research with red rectangles.

**Figure 11:**
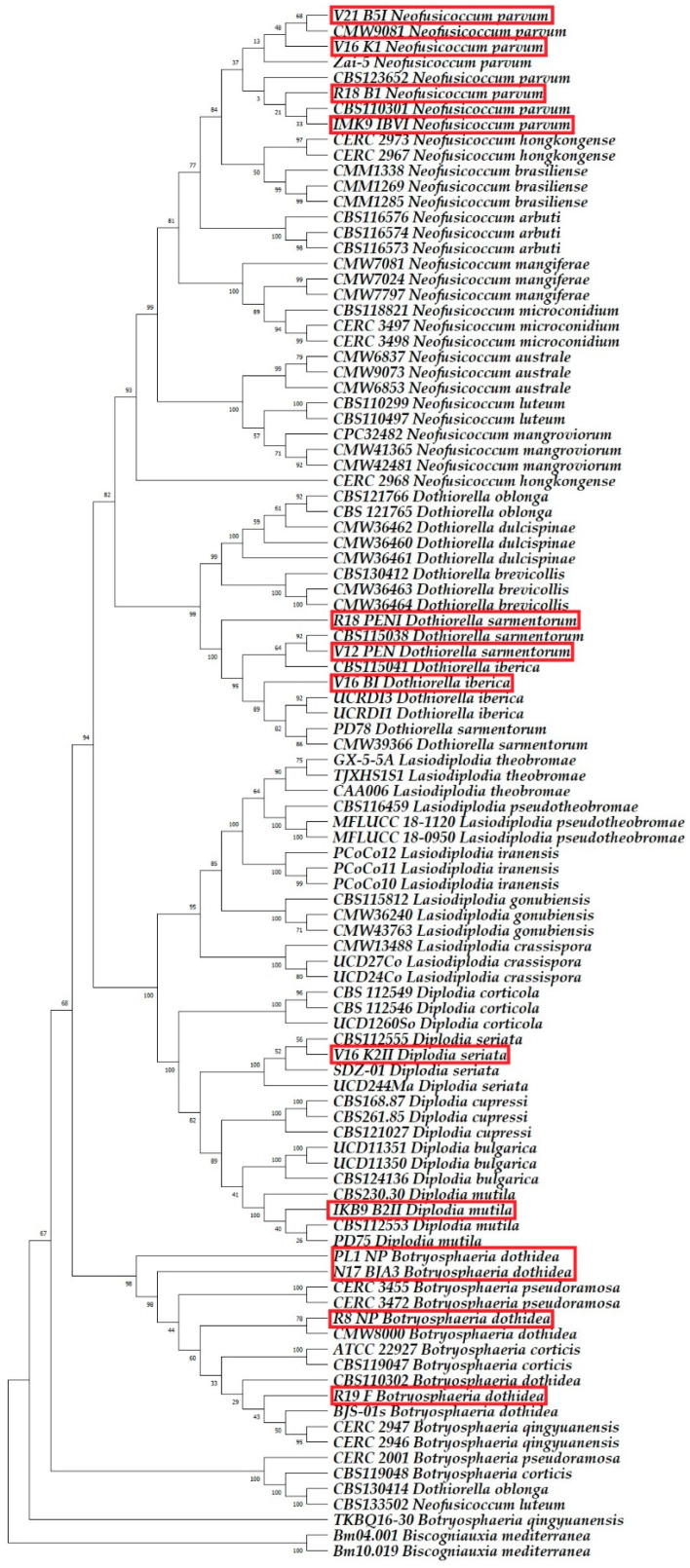
The multilocus phylogenetic tree, based on the alignment of the internal transcribed spacer, beta-tubulin, and translation elongation factor 1-alpha sequences, highlights the sequences identified in this research with red rectangles.

Phylogenetic analysis incorporated 100 nucleotide sequences per tree, with all ambiguous positions removed via the pairwise deletion option. The *Biscogniauxia mediterranea* isolates Bm04.001 and Bm10.019 were used as outgroups. The final dataset comprised 1189 positions for ITS sequence analysis, 1450 positions for *TUB2* sequence analysis, 783 positions for *TEF1-α* sequence analysis, and 2838 positions for multilocus analysis. The optimal trees are shown in Figure 8, Figure 9, Figure 10 and Figure 11.

Based on the combination of morphological characteristics, sequence analysis using the BLAST system, and phylogenetic analysis, the isolates R8 NP, PL1 NP, N17 BJA3, and R19 F were identified as *Botryosphaeria dothidea* (Moug. ex Fr.) Ces. & De Not.; isolate IKB9 B2II as *Diplodia mutila*; isolate V16 K2II as *Diplodia seriata* De Notaris; isolate V16 BI as *Dothiorella iberica* A.J.L. Phillips, J. Luque & A. Alves; isolates V12 PEN and R18 PEN1 as *Dothiorella sarmentorum* (Fr.) A.J.L. Phillips, Alves & Luque; and isolates IMK9 IBVI, V16 K1, R18 B1, and V21 B5I as *Neofusicoccum parvum* (Pennycook & Samuels) Crous, Slippers & A.J.L. Phillips.

### 2.4. Pathogenicity Test and Evaluation of Variety Resistance

#### 2.4.1. Pathogenicity Test

**Figure 12:**
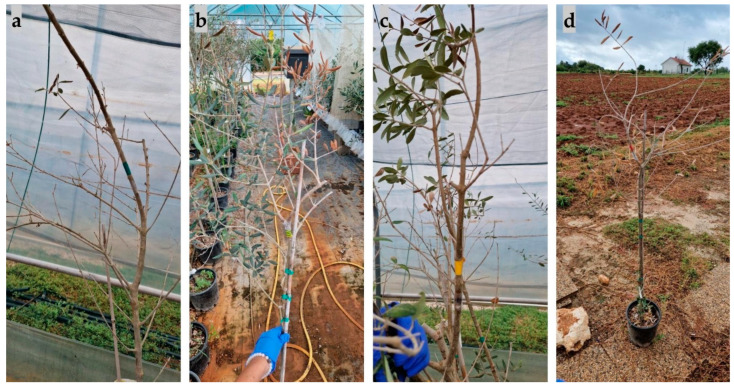
Defoliation and branch dieback symptoms on olive seedlings inoculated with (**a**) *Diplodia mutila*, (**b**) *Neofusicoccum parvum*, (**c**) *Diplodia seriata*, and (**d**) *Botryosphaeria dothidea*.

The first symptom observed on olive seedlings inoculated with species from the *Botryosphaeriaceae* family was leaf wilting, defoliation, and twig and branch dieback (Figure 12), which appeared after three months. This was most pronounced in seedlings inoculated with *B. dothidea* and *D. mutila*, and least pronounced in seedlings inoculated with *D. seriata*.

**Figure 13:**
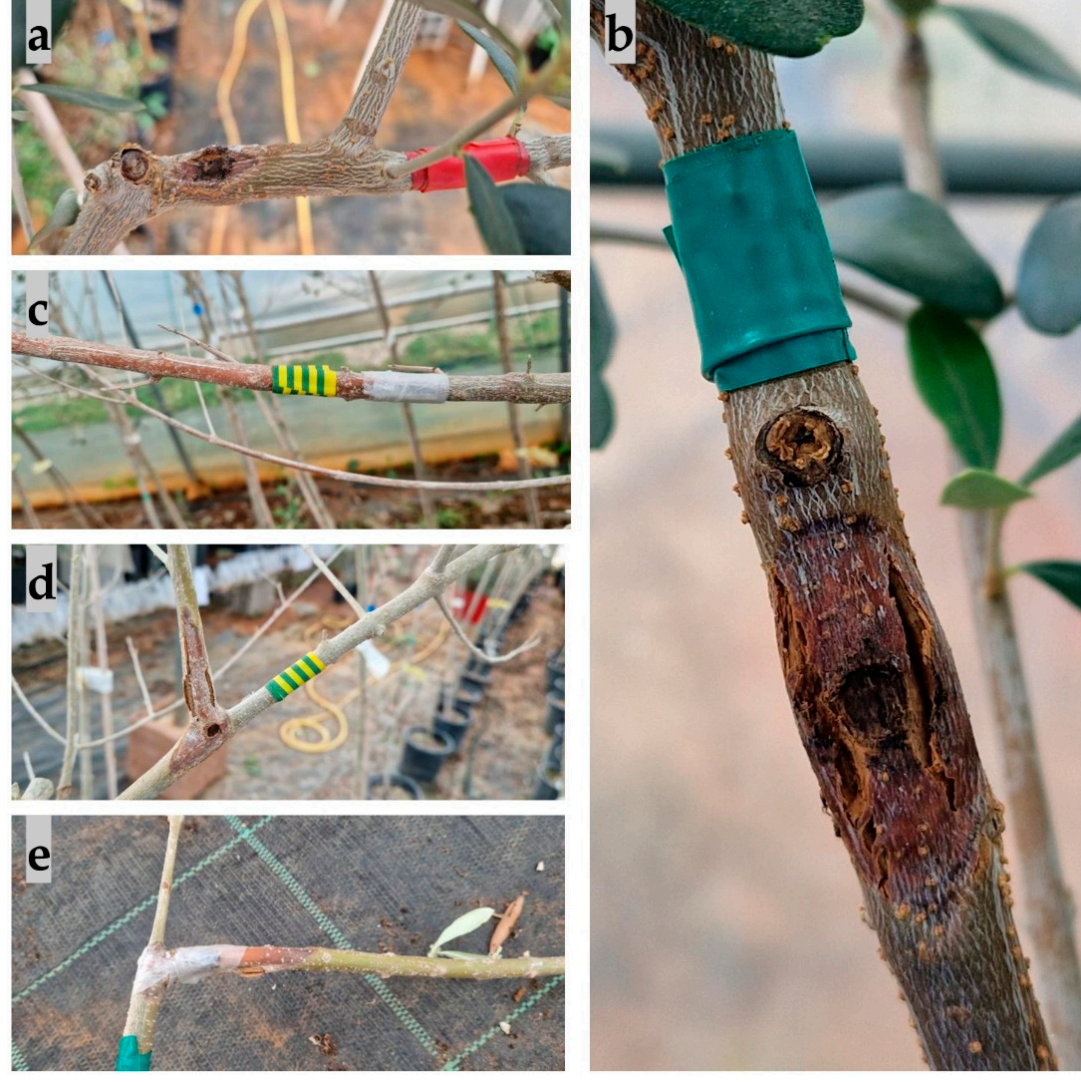
Canker formations, bark splitting and colour change on branches inoculated with the species (**a**) *Botryosphaeria dothidea*, (**b**) *Diplodia mutila*, (**c**,**d**) *Neofusicoccum parvum*, and (**e**) *Diplodia mutila*.

**Figure 14:**
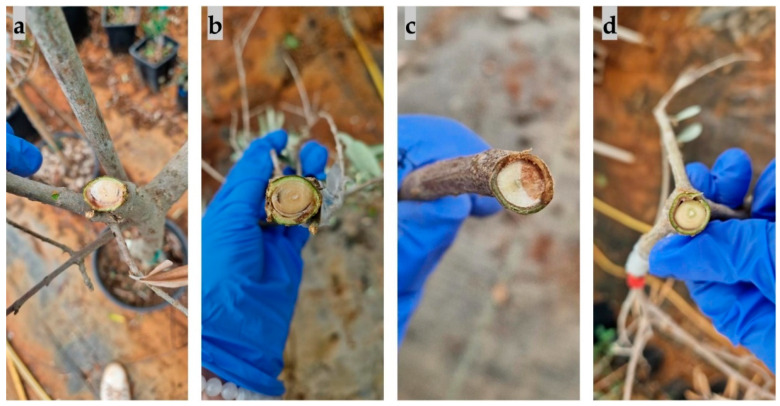
Necrosis observed in the cross-sections of branches caused by (**a**) *Diplodia mutila*, (**b**) *Dothiorella iberica*, (**c**) *Neofusicoccum parvum*, and (**d**) *Botryosphaeria dothidea*.

Other symptoms that appeared included a change in bark colour to reddish due to branch dieback, followed by the appearance of canker formations, bark cracking (Figure 13), and necrosis (Figure 14). The most aggressive species was *N. parvum*, with a total average necrotic lesion diameter of 93.45 mm. This was followed by *D. mutila*, with 33.2 mm; *B. dothidea*, with 17.8 mm; and *Do. sarmentorum*, with 11.3 mm. The least aggressive species were *Do. iberica,* with a total necrotic lesion diameter of 4.46 mm, and *D. seriata*, with 7.45 mm. For the olives inoculated with pure PDA (control group), there were no observed changes. The fungus that was re-isolated from the infected seedlings matched the originally inoculated species, thus validating Koch’s postulate.

#### 2.4.2. Variety Resistance

The olive variety Buža exhibited the highest resistance to *Do. sarmentorum*, with a statistically significant difference compared to other species (Table 6). This was followed in descending order by resistance to *Do. iberica*, *D. seriata*, and *B. dothidea*. Conversely, Buža showed the greatest susceptibility to *D. mutila* and *N. parvum*. The Istarska bjelica variety demonstrated the greatest resistance to *Do. iberica* and *Do. sarmentorum*, followed by *D. seriata* and *B. dothidea*. Its highest susceptibility was observed for *N. parvum*, with a statistically significant difference compared to other species. Istarska bjelica also showed notable susceptibility to *D. mutila*. The Leccino variety had the highest resistance to *Do. iberica* and *D. seriata*. It was most susceptible to *B. dothidea*, followed by *Do. sarmentorum* and *N. parvum*, with significant susceptibility also noted for *D. mutila*. The Rosinjola variety exhibited the greatest resistance to *Do. sarmentorum* and *Do. iberica*, followed by *D. seriata* and *B. dothidea*. It displayed significant susceptibility to *N. parvum* and *D. mutila*.

**Figure 15:**
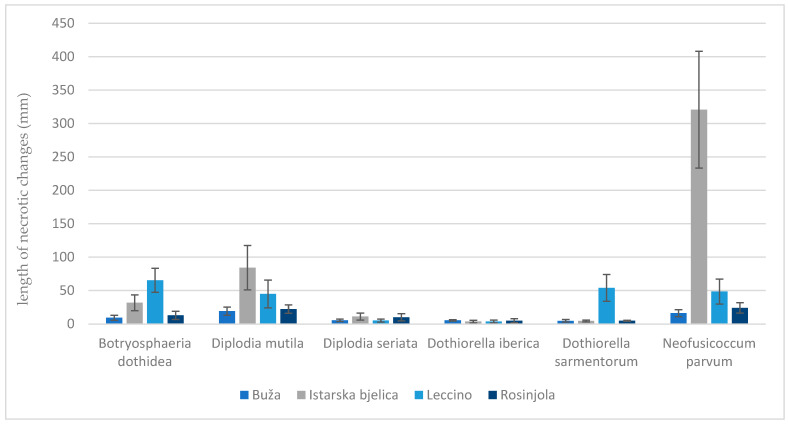
Results of pathogenicity test/variety resistance test. Average values of the length of necrotic changes (mm) per variety are shown in columns of different colours, ranging from light to dark blue. Each column represents the mean value of 10 measurements corresponding to Table 6. The vertical error bars marked in black indicate the standard deviation.

**Figure 16:**
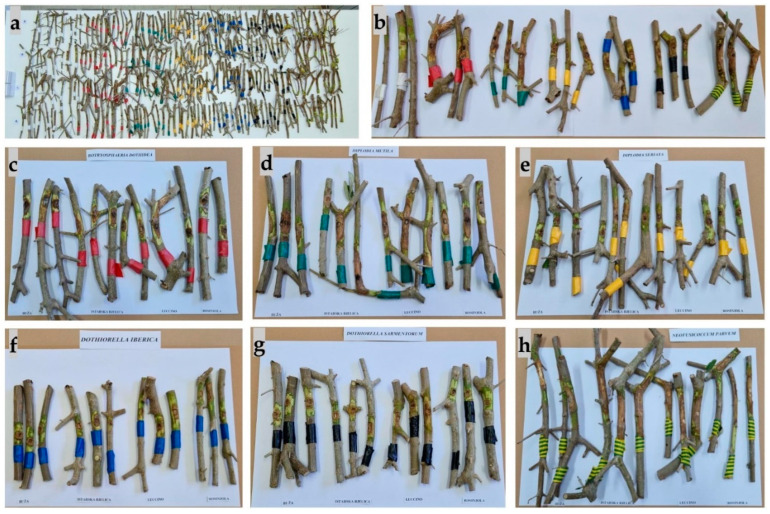
Results of pathogenicity test/variety resistance test: (**a**) samples collected from all inoculated olive seedlings; (**b**) differences between control plants and plants inoculated with species from the *Botryosphaeriaceae* family observed as follows: the white mark indicates control, the red mark indicates *Botryosphaeria dothidea*, the green mark indicates *Diplodia mutila*, the yellow mark indicates *Diplodia seriata*, the blue mark indicates *Dothiorella iberica*, the black mark indicates *Dothiorella sarmentorum*, and the yellow–green mark indicates *Neofusicoccum parvum*; (**c**–**h**) symptoms on branches per variety (from left to right: three branches from Buža, three from Istarska bjelica, three from Leccino, and three from Rosinjola): (**c**) *B. dothidea*, (**d**) *D. mutila*, (**e**) *D. seriata*, (**f**) *Do. iberica*, (**g**) *Do. sarmentorum*, (**h**) *N. parvum*.

When evaluating resistance by fungus, for *B. dothidea*, Buža demonstrated the highest resistance (9.30 ± 3.69), followed by Rosinjola (12.87 ± 6.26) and Istarska bjelica (31.75 ± 11.88), while Leccino was the most susceptible (65.41 ± 17.82). Similarly, for *D. mutila*, Buža demonstrated the highest resistance (19.16 ± 6.27), followed by Rosinjola (22.25 ± 6.36) and Leccino (44.95 ± 20.75), with Istarska bjelica being the most susceptible (84.33 ± 33.28). For *D. seriata*, Leccino demonstrated the highest resistance (5.10 ± 2.32), followed by Buža (5.50 ± 1.85) and Rosinjola (10.05 ± 5.41), with Istarska bjelica showing moderate susceptibility (11.15 ± 5.25). In the case of *Do. iberica*, Istarska bjelica was the most resistant (3.65 ± 1.93), followed by Leccino (3.75 ± 2.02) and Rosinjola (4.95 ± 2.85), while Buža was the most susceptible (5.50 ± 1.03). For *Do. sarmentorum*, Istarska bjelica also exhibited the highest resistance (4.55 ± 1.42), followed by Buža (4.70 ± 1.92) and Rosinjola (4.85 ± 0.75), with Leccino being significantly more susceptible (53.99 ± 20.04). For *N. parvum*, Buža again showed the greatest resistance (16.15 ± 5.32), followed by Rosinjola (24.11 ± 7.87) and Leccino (48.45 ± 18.69), whereas Istarska bjelica was highly susceptible (320.75 ± 87.39). This range of susceptibility highlights the varying resistance levels among different olive varieties to specific pathogenic fungi (Figure 15 and Figure 16).

## 3. Discussion

In the past, species within the *Botryosphaeriaceae* family were identified mainly by their ascospores. However, relying solely on the sexual state for classification is inadequate, particularly because some species are known only in their asexual state, and in others, the sexual state is exceedingly rare. On the other hand, conidia of the *Botryosphaeriaceae* display great variation between genera and species [37]. Two types of conidia appear: thin-walled, narrow or spindle-shaped (fusicoccum-like), and thick-walled, broader (diplodia-like) conidia [37]. *D. mutila*, *D. seriata*, *Do. iberica*, and *Do. sarmentorum* belong to the first group, as they have diplodia-like conidia, while *B. dothidea* and *N. parvum* have fusicoccum-like conidia. In this study, isolates were morphologically characterised based on the appearance of mycelium and rate of mycelial growth, as well as the appearance and dimensions of spores, and the appearance of pycnidia. These morphological characteristics aligned with those reported for species in the relevant literature [37]. According to empirical mathematical modelling, the optimal temperatures for species growth varied depending on the species and ranged between 21.3 °C and 28.1 °C. The minimum temperatures ranged between 5.1 °C and 5.4 °C, and the maximum temperatures between 31.8 °C and 39.9 °C. *N. parvum* exhibited the highest values of optimal growth temperature, while *Do. sarmentorum* exhibited the lowest. Hernandez-Rodriguez et al. [32] examined the growth rates of various *Botryosphaeriaceae* species at six temperatures (10, 15, 20, 25, 30, and 35 °C). The optimal temperature was found to be 25 °C. All isolates grew at all temperatures evaluated between 10 and 30 °C. In this study, the isolates also grew at 35 °C, but none of the isolates grew at 5 °C or 40 °C. Kaliterna [43] tested the growth rates of *B. dothidea*, *D. seriata*, *Do. sarmentorum*, and *N. parvum*. Small deviations in temperature values estimated by the empirical mathematical modelling were recorded between the mentioned study and this study. Additionally, in the mentioned study, no mycelial growth was recorded at 5 °C or 40 °C, except for *B. dothidea* at 40 °C, which ranged between 1 and 4 mm. As the author notes, the exact reason for the difference in cardinal temperatures for mycelial growth, as well as mycelial growth rates, is not fully understood, likely due to intraspecific variability among isolates.

Phillips et al. [37] consider morphological characteristics alone to be inadequate for defining genera or identifying species, given the confusion it has caused in the past, their variation during development, and inevitable overlap as representation grows. The most accurate identification of fungi is achieved through a combination of morphological characteristics of fungi and molecular methods. As a standard for the molecular identification of fungi, the ITS region of the genome is commonly used [44]. However, in recent research, additional genes such as *TUB2*, *TEF1-α*, *CALM*, *ACT*, and *COI* are also utilised [44]. As highlighted by Kaliterna [43], a drawback of such research is its high cost. In studies of species from the *Botryosphaeriaceae* family, identification is most commonly performed based on the ITS, *TUB2*, and *TEF1-α* regions of the genome [32,36,37,43]. In this study, species were molecularly identified based on the ITS, *TUB2*, and *TEF1-α* regions of the genome, as well as utilising four phylogenetic trees derived from these gene regions. A total of six species were identified on olive trees in Croatia, namely, *B. dothidea*, *D. mutila, D. seriata*, *Do. iberica*, *Do. sarmentorum*, and *N. parvum*. 

As pathogens on olives, the following species from the *Botryosphaeriaceae* family have been identified globally: *B. dothidea*; *B. wangensis* G.Q. Li & S.F. Chen; *D. africana* Damm & Crous; *D. fraxini* Fries; *D. mutila*; *D. olivarum*; *D. seriata*; *D. subglobosa* A.J.L. Phillips, Deidda & Linald; *L. theobromae* (Pat.) Griffon & Maubl.; *Do. iberica*; *Do. omnivora* Linaldeddu, Deidda & Scanu; *Do. sarmentorum*; *Do. sempervirentis* Abdollahz., Zare & A.J.L. Phillips; *N. australe*; *N. cryptoaustrale* Pavlic, Maleme, Slippers & M.J. Wingf.; *N. luteum* (Pennycook & Samuels) Crous, Slippers & A.J.L. Phillips; *N. mediterraneum*; *N. occulatum* Sakalidis & T. Burgess; *N. parvum*; *N. ribis*; *N. vitifusiforme*; and *Sardiniella urbana* Linaldeddu, A. Alves & A.J.L. Phillips [16,32,35,36,45,46,47,48]. In Croatia, three species from the *Botryosphaeriaceae* family have been identified as pathogens on olive trees: *B. dothidea*, *D. seriata*, and *N. parvum* [15,16,17,18]. It is known that species from this family attack numerous plant species, predominantly woody ones, such as grapevine (*V. vinifera*) [49], European beech (*Fagus sylvatica* L.) [50], plum (*Prunus salicina* Lindl.) [51], etc. In Croatia, besides on olive trees, they have also been found on grapevine (*V. vinifera*) [43], walnut (*Juglans* sp.) [52], and giant sequoia (*Sequoiadendron giganteum* (Lindl.) J. Buchholz) [53]. On grapevines, the species *B. dothidea*, *D. coryli*, *D. seriata*, *Do. sarmentorum*, and *N. parvum* have been identified [43]. On walnut trees, *B. dothidea* and *N. parvum* have also been identified, while in giant sequoia, *B. dothidea* and *N. yunnanense* G.Q. Li & S.F. Chen have been recorded [52,53].

Various other fungi described in our previous studies were also identified at some locations. In grove R18 (Table 1), the fungal species *B. mediterranea* [19], *N. philosophiae-doctoris* [22], and *P. iranianum* [23] were identified, along with *Do. sarmentorum* and *N. parvum* detected in this study. In grove V16, fungi including *B. nummularia* (Bull.) Kuntze [19] and *C. pruinosa* [21] were identified, alongside *D. seriata*, *Do. iberica*, and *N. parvum*. In grove IMK9, the fungi *B. mediterranea* [19] and *N. parvum* were identified, while in grove N17 and R19, the species *B. mediterranea* [19] and *B. dothidea* were found. Mutual infection was not observed on all sampled trees. In this research, the species *B. dothidea* and *N. parvum* were the most frequently found *Botryosphaeriaceae* species on olive trees. In the study by Linaldeddu et al. [48], fungi from the *Botryosphaeriaceae* family were the main species isolated from olive trees with branch cankers and fruit rots. Similarly, Hernandez-Rodriguez et al. [32] predominantly isolated species from the genera *Botryosphaeria* (41%) and *Neofusicoccum* (51%) from olives, with *Diplodia* being less common (8%). In the study by Lazzizera et al. [33], *Botryosphaeria* and *Neofusicoccum* species were isolated from over 60% of the affected drupes, indicating they are the primary contributors to the disease. The most frequently isolated species was *B. dothidea*, found in 34% of the drupes. *B. dothidea* ranks among the most globally prevalent species [32]. The prevalence and distribution of these species may be influenced by climatic and geographical factors, which could be particularly pronounced in Croatia, as noted by Kaliterna [43]. When the climate conditions are optimal, phytopathogenic fungus can grow exponentially and devastate crops, which can cause high economic losses [54]. Humans and animals can also suffer the consequences of fungi attacks because of the mycotoxins produced by some fungi.

Most fungal species responsible for causing fruit rot in olives are typically common saprophytes or secondary invaders that usually enter through wounds inflicted by biotic or abiotic factors. Fungi of the *Botryosphaeriaceae* family have been recognised as some of the most aggressive and prevalent pathogens impacting olives in olive cultivation areas like California, Italy, South Africa, and others [16,32,35,36]. Different fungi can attack different plant organs, so fungal infections cause an enormous range of disease symptoms, such as colour and shape changes, rotting, wilting, and wounds. Cell death causes parts of the plant to decompose and turns plant tissues into a dark colour; this can appear as spots on leaves or rotten spots on fruits [54]. According to the literature, species from the *Botryosphaeriaceae* family cause symptoms that include fruit rot, leaf wilting and defoliation, branch and twig dieback, canker formation, and necrosis of internal tissue, as well as discolouration of the bark, among others [16,32,35,36]. These symptoms were also observed in this research, both in the field and on olive seedlings following pathogenicity tests.

In research conducted by Moral et al. [31], pathogenicity tests using unripe olive fruit and olive branches showed that *D. seriata* isolates were the least aggressive on both the fruit and branches, while *N. mediterraneum* isolates were the most aggressive in both tissues. Isolates of *B. dothidea* were not pathogenic on branches and only weakly aggressive on fruit. The most aggressive species in our research were *N. parvum*, *D. mutila*, and *B. dothidea*, whereas the least aggressive species was *Do. iberica*. As stated by Hernandez-Rodriguez et al. [32] in their studies, the *Neofusicoccum* isolates were also considerably more aggressive than the *Botryosphaeria* and *Diplodia* isolates. The same case was recorded in the pathogenicity tests in the study by Linaldeddu et al. [48], where *N. parvum* caused much larger lesions than species *B. dothidea*, *D. fraxini*, *D. mutila*, *D. olivarum*, and *D. subglobulosa*. Contrary to that, in the study by Godena et al. [17], *D. seriata* proved to be more aggressive than *N. parvum*.

The disease resistance of olive varieties offers an economically feasible alternative to chemical control, with minimal environmental impact, and can be integrated into pest management strategies [46]. Theophrastus [2] observed that the Greeks preferred to propagate olives through cuttings, knowing from experience that olives grown from seeds were inferior, thus requiring improvement through grafting and thereby creating more resistant trees. As a measure against *B. dothidea*, the use of varieties resistant to olive fruit fly attack may serve (since the parasite of the fly, *L. berlesiana*, cannot penetrate the fruit on its own), along with monitoring the fly occurrence using traps with attractants [46]. Varieties identified as more susceptible to olive fruit fly infestation include St. Catarina and Ascolara Tenera, while varieties such as Dužica [7], Nocellara etnea, Oliva di Cerignola, Orbetana, and Capolga have shown resistance [4]. As a preventive measure, varieties more resistant to disease-causing agents can be planted. Latinović et al. [30] tested the resistance of 17 olive varieties to *B. dothidea*. The most resistant were Crnjaka and Gloginja, along with Pendolino and Cassanesse. Moderately resistant varieties included Picholine, Grossa di Spagna, and Conserviola, while Rogganiella, Lumbardeška, Sant Agostino, Manzanilla, and Noccelara del Belice were rated as intermediate. Leccino, Coratina, and Žutica showed moderate susceptibility, while Giarraffa and Ascolana tenera were highly susceptible. Moral et al. [46] tested the resistance of the 11 most important table varieties to *N. mediterraneum* and *B. dothidea*. Testing results on branches showed that Gordal Sevillana, followed by Santa Caterina and San Agostino, were the most susceptible to *N. parvum*. Manzanilla Cacereña was the most resistant, followed by Verdial de Huévar and Morona. Concerning potted plant inoculation with *N. mediterraneum*, Manzanilla Cacereña and Gordal Sevillana were the most susceptible, while the most resistant were Verdial de Huévar, Hojiblanca, and Aloreña de Atarfe. The results of testing resistance on olive fruit showed that the most resistant varieties to *B. dothidea* were San Agostino and Hojiblanca, while Aloreña de Atarfe was the most susceptible. According to the authors, detached branches may experience significant stress and may not behave physiologically in the same manner as branches attached to a tree. Therefore, results obtained from detached branches may not be entirely indicative of varieties’ resistance. In this study, the susceptibility of varieties varied depending on the fungal species with which the olive seedlings were inoculated. When evaluating resistance by fungus, for *B. dothidea*, Buža demonstrated the highest resistance, while Leccino was the most susceptible. For *D. mutila*, Buža showed the highest resistance, whereas Istarska bjelica was the most susceptible. In the case of *D. seriata*, Leccino exhibited the highest resistance, with Istarska bjelica being most susceptible. For *Do. iberica* and *Do. sarmentorum*, Istarska bjelica showed the highest resistance, while Buža and Leccino were the most susceptible, respectively. For *N. parvum*, Buža had the highest resistance, while Istarska bjelica was highly susceptible. An important factor is not only the resistance of varieties but also the timing of fruit ripening, i.e., the occurrence of apparent resistance. The fruits of most varieties ripen between November and February, and the moment they must be harvested depends on the olive tree’s location, exposure, and meteorological conditions [4]. Early-ripening varieties harvested in late September and October avoid the most intense periods of olive fruit fly attacks [7]. Therefore, when establishing an olive grove, it is advisable to arrange varieties in separate rows to facilitate harvest segregation based on genotype and relative ripening period [4]. As noted by Latinović et al. [30], the identification of resistant varieties could represent the fundamental element of cost-effective disease management, especially for numerous small-scale growers unable to afford pesticide spraying for large olive trees.

Besides resistant varieties, disease control strategies include the removal of infected plant parts [55]. Pruning should be conducted during dry weather with clean and disinfected tools, and coating wounds after pruning is also crucial. Pitt et al. [56] and Díaz and Latorre [57] report that treating wounds with fungicides and horticultural wax helps reduce the incidence of Botryosphaeria dieback. However, it is important to carefully select the fungicide to be applied, as in vitro studies have shown that not all fungicides are equally effective against all species of this family [56,58]. Among the most effective fungicides are those containing the active ingredients benomyl, carbendazim, fluazinam, flusilazole, fludioxonil, iprodione, myclobutanil, penconazole, procymidone, pyraclostrobin, thiophanate–methyl, and tebuconazole [55,56]. When establishing an olive grove, it is also essential to consider the choice of location.

## 4. Materials and Methods

### 4.1. Fieldwork and Isolation of Fungi

**Figure 17:**
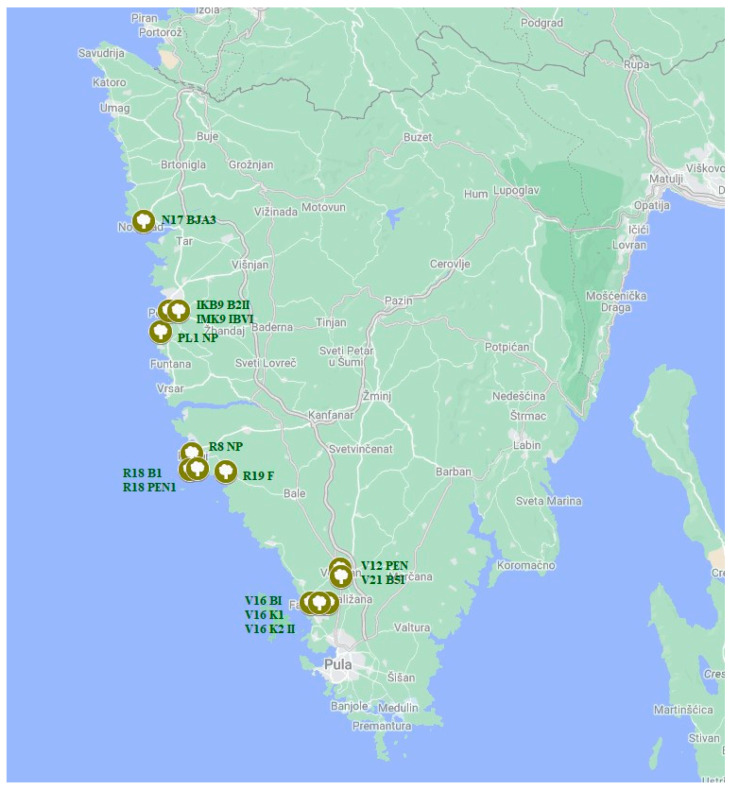
Locations of samples of species from the *Botryosphaeriaceae* family collected from olive trees in Istria, Croatia, are marked with green–white labels, with the isolate names next to the labels. The figure was made using Google Maps.

As part of the olive disease research conducted in Istria County in 2021 and 2022, a total of 26 locations were surveyed, and samples were collected from a total of 112 olive trees. The Botryosphaeria dieback of olive was confirmed at 10 of these locations (Figure 17), i.e., the disease was observed on a total of 13 trees.

Information regarding the precise location and coordinates of the site where species from the family *Botryosphaeriaceae* were found, including the variety from which the sample was taken, collection date, olive grove area, and tree age, are presented in Table 7. 

The trees exhibited symptoms such as leaf wilting and defoliation, twig and branch dieback, and the appearance of necrosis and cankers. A total of 10 branch samples per tree were collected. Samples were taken from the parts of the branches where the transition between healthy and infected parts were visibly apparent. The samples were placed in sterile black plastic bags, labelled, and stored in a portable refrigerator at a temperature of +4 °C. The collected samples were promptly transported to the Laboratory for Plant Protection at the Institute of Agriculture and Tourism in Poreč, Croatia, for analysis. Branch samples from affected trees were photographed and documented and then underwent a washing under tap water. With the use of a sterile surgical scalpel, the bark was removed from the branches, and subsequently, the samples were cut using fruit shears. The branch pieces (5 × 5 cm) were immersed in 70% ethanol for two minutes, followed by rinses in sterile distilled water for two minutes. After this process, they were carefully arranged on a sterile paper sheet within a laminar flow cabinet to facilitate surface drying. Once adequately dried, the pieces were placed on PDA supplemented with 35 mg/L of penicillin and incubated in a dark environment at 25 °C within an incubator. Upon the development of the culture on PDA, isolates were transferred to a medium containing WA and pine needles (*Pinus* L.) (WA + *Pinus*). The medium preparation involved cutting fresh green pine needles, washing them under tap water, and autoclaving them twice in a glass jar at 121 °C for 15 min. Two to three pieces of pine needles were placed on the surface of WA after the agar had solidified by 50%, submerging half of the needle in the medium while leaving the rest exposed. Fungal isolates were then inoculated into the nutrient medium WA + *Pinus*. After an incubation period of 20 days and the subsequent development of pycnidia, spores were extracted to create single-spore isolates. Pure cultures were preserved in 2 mL cryovial screw cap tubes containing a 50% glycerol solution at temperatures of −20 °C and −80 °C, as well as in sterilised water in plastic tubes at 4 °C. The preserved cultures are kept in the Laboratory for Plant Protection collection at the Institute of Agriculture and Tourism in Poreč.

### 4.2. Morphological Characterisation

Following incubation at 25 °C in the absence of light for 2, 15, and 30 days, pure fungal cultures were subjected to examination. The preliminary determination involved an inspection of colonies, considering their overall appearance and colour. Additionally, the observation of conidia included an assessment of colour, appearance, septation, and shape. Out of the 10 samples collected per tree (totalling 13 trees), the identical fungus was consistently isolated. For a detailed analysis of morphological characteristics, one representative isolate per tree was selected, and a total of 13 fungal isolates were analysed. This involved a detailed evaluation of colony traits, encompassing parameters such as colour, shape, elevation, margin, surface, and opacity. Macroscopic characteristics were observed using a BOECO zoom stereo microscope BSZ-405 and photographed with a fitted B-CAM16 industrial digital camera and B-View software (Boeckel, Hamburg, Germany) at the Institute of Agriculture and Tourism Poreč, as well as with an Olympus SZX10 microscope and Olympus N547 camera (Olympus, Tokyo, Japan) at the Faculty of Agrobiotechnical Sciences Osijek. Additionally, the determination of growth rate and cardinal temperatures for mycelial growth was performed. To determine the cardinal temperatures for growth, five-day-old isolates were inoculated in triplicate on PDA in Petri dishes with a diameter of 90 mm. A circular section of mycelium with a diameter of five mm was placed at the centre of the PDA. The isolates were incubated in darkness at eight different temperatures (5, 10, 15, 20, 25, 30, 35, and 40 °C), and measurements were taken after 48 h. Colony diameter was measured at two positions perpendicular to each other, and the obtained values were reduced by the diameter of the circular section of the mycelium. The average values were then calculated. Empirical mathematical modelling, employing the least squares method, as described by Sanchez et al. [59], was conducted within the suitable frameworks of third, fourth, fifth, and sixth-degree polynomial regressions using Microsoft Office Excel. This approach was used to generate graphs along with corresponding equations, serving to determine cardinal temperatures. Since the sixth-degree polynomial equation fits the examined data best and closely follows the points of determined growth rates, it was utilised for further determination of cardinal temperatures. In order to obtain the minimum, maximum, and optimal cardinal temperatures from the equation, the described empirical mathematical modelling using the least squares method was performed with the Wolfram Alpha (WolframAlpha LLC) mathematical program.

Furthermore, the features of spores, including characteristics such as colour, shape, the presence or absence of septum, and dimensional measurements, were examined. To determine the morphological characteristics of the conidia, they were extracted from pycnidia cut with a laboratory needle. Microscopic analysis was conducted using an LABOSGEN camera and LABOSGEN software at the Institute of Agriculture and Tourism Poreč, as well as with an Olympus BX41 microscope (Olympus, Tokyo, Japan) at the Faculty of Agrobiotechnical Sciences Osijek. Additionally, measurements of 30 conidia per isolate were performed. Subsequently, mean values, standard deviations of measured conidia and their minimum and maximum values, and the length-to-width ratio of conidia were calculated. Furthermore, 95% confidence intervals for the determined mean dimensions and length-to-width ratio were established using Microsoft Office Excel. The morphological profile derived from this analysis was systematically compared with relevant scientific literature sources [37].

### 4.3. DNA Extraction and Amplification

Molecular analysis was used to confirm the identification of all 13 isolates at the species level. Fungal isolates were cultured on PDA for seven days at 25 °C in the dark. Subsequently, a small portion of mycelium from the colony margins was aseptically sampled using a sterile laboratory needle for genomic DNA extraction. Maxwell^®^ RSC Instrument (Promega, Madison, WI, USA) and Maxwell^®^ RSC Plant DNA Kit (Promega, Madison, WI, USA) were used to extract total genomic DNA. The amount of genomic DNA in samples post-isolation was quantified using a Maxwell Promega Quantus fluorometer (Promega). The internal transcribed spacer (ITS) regions were subjected to amplification and subsequent sequencing using the primer pairs ITS1 (5′ TCCGTAGGTGAACCTGCGG 3′) and ITS4 (5′ TCCTCCGCTTATTGATATGC 3′) [60]. Amplification of a segment of the beta-tubulin (*TUB2*) gene was carried out utilising the oligonucleotide primers Bt2a (5′ GGTAACCAAATCGGTGCTGCTTTC 3′) and Bt2b (5′ ACCCTCAGTGTAGTGACCCTTGGC 3′) [61]. Furthermore, a part of the translation elongation factor 1-alpha gene (*TEF1-α*) was amplified and sequenced using the primer pairs EF-728F (5′ CATCGAGAAGTTCGAGAAGG 3′) and EF1-986R (5′ TACTTGAAGGAACCCTTACC 3′) [62]. Each PCR mixture, with a final volume of 25 µL, was composed of 12.5 µL of EmeraldAmp^®^ GT PCR Master Mix, 0.5 µL of each primer (10 µM), 6.5 µL of nuclease-free water, and 5 µL of template DNA at a concentration of 5 ng/µL. PCR amplification was performed using a SureCycler 8800 Thermal Cycler (Agilent Technologies, Santa Clara, CA, USA). The amplification program comprised an initial denaturation step at 94 °C for two minutes, followed by 40 cycles of denaturation at 94 °C for 30 s, annealing at 55 °C for 45 s, elongation at 72 °C for one minute and 30 s, and a final extension step at 72 °C for five minutes [63]. Gel electrophoresis was performed utilising a 1% agarose gel at 110 V for 25 min in 1× TAE buffer, employing an omniPAC Midi CS-300V electropshoresis power supply (Cleaver Scientific, Rugby, Warwickshire, UK). Following electrophoresis, visualisation of PCR products was accomplished using an iBright CL1000 Imaging System (Invitrogen, Thermo Fisher Scientific, Waltham, MA, USA). After visualisation, purification of the PCR products was carried out using the GenElute™ PCR Clean-Up Kit (Sigma-Aldrich, Burlington, MA, USA).

### 4.4. DNA Sequence Assembly and Phylogenetic Analysis

Sequencing of the PCR products was carried out by the Macrogen Europe sequencing service (Amsterdam, the Netherlands). Sequencing was performed bidirectionally using the same primers that were used for amplification. Subsequently, nucleotide sequences were read and edited using Sequencher^®^ software (Gene Codes Corporation, Ann Arbor, MI, USA). Comparative analysis was conducted against existing sequences from the *Botryosphaeriaceae* family available in the National Center for Biotechnology Information GenBank database. Consensus sequences resulting from this study were submitted to NCBI GenBank. Phylogenetic analyses were conducted for individual gene regions as well as for combined regions. Sequence data from isolates used in this study and relevant isolates from GenBank were utilised for phylogenetic analysis. The list of species/isolates included in the phylogenetic analysis is represented in Table 8. Sequence alignment was performed using ClustalX2 software (UCD, Dublin, Ireland) using the following multiple alignment parameters: gap opening = 15, gap extension = 6.66, delay divergent sequences = 30%, DNA transition weight = 0.5. The evolutionary history was deduced utilising the neighbour–joining method [64], with the optimal tree depicted. Bootstrap analysis (1000 replicates) indicated the percentage of replicate trees in which the associated taxa clustered together, shown next to the branches [65]. The evolutionary distances were calculated using the maximum composite likelihood method and are presented as the number of base substitutions per site [66]. MEGA11 (Pennsylvania State University, State College, PA, USA) was employed for the evolutionary analyses [67].

### 4.5. Pathogenicity Test and the Evaluation of Variety Resistance

For confirmation of species pathogenicity and evaluation of variety resistance to identified species, a greenhouse experiment was set up using olive seedlings. One representative isolate of each *Botryosphaeriaceae* species (a total of six isolates) identified in this study was selected for pathogenicity testing. Since multiple isolates were collected for certain species while only one for others, isolates with similar growth rates on PDA were chosen. The experiment utilised five-year-old seedlings of three Croatian indigenous olive varieties: Buža, Istarska bjelica, and Rosinjola, as well as one introduced variety: Leccino. The bark at the intended inoculation site was wiped with cotton soaked in 70% ethanol. Wounds measuring five mm in diameter were then created using a sterile cork borer. The outer bark was removed while preserving the inner bark. A 5 mm-diameter mycelium plug from a 10-day-old colony on PDA was inserted into the wound using a sterile cork borer. Inoculated wounds were coated with Vaseline and covered with Parafilm. Pure PDA plugs served as controls. Ten seedlings were inoculated per isolate. The inoculated plants were grown in the greenhouse for nine months, from December 2022 to October 2023. The seedlings were irrigated using a drip irrigation system. The average temperature during the experimental period in the greenhouse ranged between 24 and 25 °C, with a relative humidity of 85%. Changes were recorded over time. After the incubation period, samples were collected in black plastic bags, and the total length of surface necrotic changes above and below the inoculation site was measured. In accordance with Koch’s postulates, small necrotic tissue fragments from the periphery of lesions that had developed on each seedling were inoculated onto PDA medium to isolate the originally introduced fungus. The data obtained from the pathogenicity assay underwent analysis of variance (ANOVA), followed by Tukey’s test to identify significant differences between mean values at a significance level of 5% [43]. Statistical analysis was conducted using the SAS Enterprise Guide 8.4 statistical software.

## 5. Conclusions

In conclusion, six different species have been identified as causative agents of Botryosphaeria dieback of olive in Istria: *Botryosphaeria dothidea*, *Diplodia mutila*, *D. seriata*, *Dothiorella iberica*, *Do. sarmentorum*, and *Neofusicoccum parvum*. To our knowledge, *D. mutila*, *Do. iberica*, and *Do. sarmentorum* have not been previously identified in olive trees in Croatia, making this the first report of their presence. Species from the *Botryosphaeriaceae* family are economically significant pathogens due to their detrimental impact on olive trees, causing fruit rot, leaf wilting, necrosis, and other symptoms. They rank among the most aggressive pathogens attacking olive trees. Therefore, it is necessary to monitor olive groves and track the further movement of these pathogens to minimise the damage they cause. Preventive measures are of utmost importance in controlling the further spread of these pathogens. These measures include disinfection of tools, pruning of olive trees and burning of residues, selection of planting locations, selection of resistant varieties, etc. The varieties tested in this study showed differences in resistance depending on the fungus with which they were infected. In addition to preventive measures, it is important to protect olive groves by using preparations that have proven effectiveness against these pathogens.

## Figures and Tables

**Table 1:** Compilation of production methods, tree density, fertilisation practices, cultivation techniques, pre-culture, surrounding vegetation, and harvesting methods in olive groves.

Location *	Production Method **	Tree Density	Fertilisation ***	Cultivation ****	Pre-Culture	Surrounding Vegetation	Harvesting Method
R8	O	2 × 3	NA	UC	Low shrubbery	Olive grove	Manual
IKB9	C	6 × 6	CAN	C	Grains, vineyards, forage crops	Olive grove	Handheld shakers
IMK9	C	6 × 6	CAN	C	Grains, vineyards, forage crops	Vineyards, forest	Handheld shakers
V12	C	5 × 5	NA	UC	Forest, meadow	Olive grove	Tractor-mounted shakers
V16	O	6 × 6 and 8 × 8	NA	C	Fruit trees	Olive grove, forest	Handheld shakers
N17	C	6 × 7 and 5 × 6	CAN	UC	Low shrubbery, meadow	Vineyards, forest	Manual, handheld shakers
R18	C	6 × 6	CAN	C	Vineyards	Meadow, field, forest	Handheld shakers
R19	C	6 × 6	Stallatico	C	Vineyards	Olive grove, vineyards, meadow, forest	Manual, handheld shakers
PL1	C	10	NA	UC	Low shrubbery, meadow	Olive trees located by the sea, surrounded by a forest	Manual
V21	C/I	6 × 6, 5 × 7 and 7 × 7	Sheep manure, compost	C/UC	Olive	Olive grove	Handheld shakers

* Location: The location tags are identical to the first half of the isolate names. ** C—conventional, I—integrated, O—organic. *** NA—not applicable, CAN—calcium ammonium nitrate. **** C—cultivated, UC—uncultivated.

**Table 2:** Conidial dimensions of fungal isolates.

Species	Conidial Size (µm)		
	Length × Width *	Average ± SD **	95% Conf. ***
*Botryosphaeria dothidea*	(20.0) 22.9–24.2 (27.3) ± (5.1) 6.0–6.6 (7.8)	23.5 ± 1.9 × 6.3 ± 0.8	0.7–0.3
*Diplodia mutila*	(20.4) 23.5–24.8 (27.4) ± (9.1) 10.8–11.6 (14.2)	24.1 ± 1.8 ×11.2 ± 1.1	0.6–0.4
*Diplodia seriata*	(16.2) 22.1–23.7 (27.6) ± (8.3) 10.7–11.7 (13.9)	22.9 ± 2.1 × 11.2 ± 1.3	0.8–0.5
*Dothiorella iberica*	(15.7) 20.4–21.7 (23.9) ± (7.1) 8.4–9.1 (10.7)	21.0 ± 1.8 × 8.7 ± 0.9	0.7–0.4
*Dothiorella* *sarmentorum*	(15.5) 18.9–20.1 (22.9) ± (7.3) 8.3–8.9 (10.9)	19.5 ± 1.7 × 8.6 ± 0.9	0.6–0.3
*Neofusicoccum parvum*	(9.2) 11.7–12.6 (15.5) ± (3.6) 5.4–6.0 (7.1)	12.2 ± 1.3 × 5.7 ± 0.9	0.5–0.3

* Dimensions are expressed as a range from the lower to the upper limit of the 95% confidence interval, with the minimum and maximum values of the measured dimensions shown in parentheses. ** Mean dimension values (length × width) and standard deviation (SD) are presented. *** The length-to-width ratio of conidia is depicted as a range from the lower to the upper limit of the 95% confidence interval.

**Table 3:** The average mycelial growth rate (mm) after 48 h, incubated at eight different temperatures.

Temperature (°C)	*Botryosphaeria dothidea*	*Diplodia mutila*	*Diplodia seriata*	*Dothiorella iberica*	*Dothiorella sarmentorum*	*Neofusicoccum parvum*
	Growth of Mycelium (mm)					
5	0	0	0	0	0	0
10	2.58	5.16	8.5	11.0	18.33	7.03
15	8.87	12.0	19.16	23.5	38.0	10.21
20	24.29	33.3	45.33	45.66	57.0	28.16
25	39.04	48.33	55.0	50.33	57.41	40.95
30	30.17	31.16	44.66	7.16	16.83	51.95
35	16.47	1.83	4.16	0	0	5.04
40	0	0	0	0	0	0

**Table 4:** The cardinal temperature for mycelium isolate growth derived from empirically determined growth rate values and mathematical modelling outcomes.

Species	Cardinal Temperatures (°C) for Mycelial Growth Based on Empirically Determined Growth Rate Values			Cardinal Temperatures (°C) for Mycelial Growth Estimated through Mathematical Modelling		
	Minimum	Optimal	Maximum	Minimum	Optimal	Maximum
*Botryosphaeria dothidea*	5–10	25	35–40	5.1	25.7	39.9
*Diplodia mutila*	5–10	25	35–40	5.1	24.9	35.3
*Diplodia seriata*	5–10	25	35–40	5.1	25.4	35.6
*Dothiorella iberica*	5–10	25	30–35	5.1	26.9	31.8
*Dothiorella sarmentorum*	5–10	25	30–35	5.2	21.3	34.3
*Neofusicoccum parvum*	5–10	30	35–40	5.4	28.1	35.5

**Table 5:** List of accession numbers of isolates deposited in GenBank.

Isolate	Species	GenBank Accession Number		
		ITS	*TUB2*	*TEF1-α*
R8 NP	*Botryosphaeria dothidea*	OQ338370	OQ348378	OQ348385
PL1 NP	*Botryosphaeria dothidea*	OQ352832	OQ361692	OQ553927
N17 BJA3	*Botryosphaeria dothidea*	OQ353073	OQ361697	OQ553926
R19 F	*Botryosphaeria dothidea*	OQ354201	OQ361700	OQ361701
IKB9 B2II	*Diplodia mutila*	OQ338569	OQ348379	OQ348386
V16 K2II	*Diplodia seriata*	OQ352870	OQ361695	OQ361696
V16 BI	*Dothiorella iberica*	OQ339205	OQ348381	OQ348388
V12 PEN	*Dothiorella sarmentorum*	OQ339150	OQ348380	OQ348387
R18 PEN1	*Dothiorella sarmentorum*	OQ341230	OQ348383	OQ348390
IMK9 IBVI	*Neofusicoccum parvum*	OQ352837	OQ361693	OQ361694
V16 K1	*Neofusicoccum parvum*	OQ341191	OQ348382	OQ348389
R18 B1	*Neofusicoccum parvum*	OQ353087	OQ361698	OQ361699
V21 B5I	*Neofusicoccum parvum*	OQ341428	OQ348384	OQ553928

**Table 6:** The results of the pathogenicity test/variety resistance test with average values of the length of necrotic changes (mean ± standard deviation, in mm) of six isolates of fungi from the *Botryosphaeriaceae* family on olive seedlings, with sterile PDA as a negative control.

Species	Variety *			
	Buža	Istarska Bjelica	Leccino	Rosinjola
*Botryosphaeria* *dothidea*	9.30 ± 3.69 b	31.75 ± 11.88 c	65.41 ± 17.82 a	12.87 ± 6.26 b
*Diplodia* *mutila*	19.16 ± 6.27 a	84.33 ± 33.28 b	44.95 ± 20.75 b	22.25 ± 6.36 a
*Diplodia* *seriata*	5.50 ± 1.85 b	11.15 ± 5.25 c	5.10 ± 2.32 c	10.05 ± 5.41 bc
*Dothiorella* *iberica*	5.50 ± 1.03 b	3.65 ± 1.93 c	3.75 ± 2.02 c	4.95 ± 2.85 cd
*Dothiorella* *sarmentorum*	4.70 ± 1.92 bc	4.55 ± 1.42 c	53.99 ± 20.04 ab	4.85 ± 0.75 cd
*Neofusicoccum* *parvum*	16.15 ± 5.32 a	320.75 ± 87.39 a	48.45 ± 18.69 ab	24.11 ± 7.87 a
Control	0.0 ± 0.0 c	0.0 ± 0.0 c	0.0 ± 0.0 c	0.0 ± 0.0 d
Minimum significant difference	4.87	48.62	19.99	6.90

* Mean values with the same letters in a row are not significantly different, according to Tukey’s honestly significant difference test (*p* < 0.05).

**Table 7:** Isolate label, location, and coordinates of *Botryosphaeriaceae* species site, olive variety from which the sample was taken, collection date, olive grove area, and tree age.

Isolate	Location	Coordinates	Variety from Which Sample Was Taken	Collection Date	Olive Grove Area (Ha)	Tree Age (Years)
R8 NP	Rovinj	45°05′20″ N, 13°38′51″ E	Unknown	6 September 2021	0.01	15
IKB9 B2II	Poreč	45°13′19.9″ N, 13°36′07.7″ E	Buža	13 September 2021	1.49	>30
IMK9 IBVI	Poreč	45°13′13″ N 13°36′09.8″ E	Istarska bjelica	13 September 2021	1.49	>30
V12 PEN	Vodnjan	44°57′65″ N, 13°50′19″ E	Pendolino	24 September 2021	0.1	20–100
V16 BI	Fažana near Vodnjan	44°56′21″ N; 13°50′18″ E	Buža	14 October 2021	1	13
V16 K1			Karbonaca			
V16 K2II			Karbonaca			
N17 BJA3	Novigrad	45°20′08.8″ N, 13°33′33.6″ E	Istarska bjelica	14 October 2021	3	20–25
R18 B1	Rovinj	45°03′02.2″ N 13°42′43.9″ E	Buža	14 October 2021	0.43	39
R18 PEN1			Pendolino			
R19 F	Rovinj	45°03′46″ N, 13°42′71″ E	Frantoio	14 October 2021	0.38	25–30
PL1 NP	Poreč	45°12′26″ N 13°35′29″ E	Unknown	23 March 2022	0.02	10
V21 B5I	Vodnjan	44°57′34″ N, 13°50′37″ E	Buža	24 March 2022	8.2	12–300

**Table 8:** List of species/isolates included in the phylogenetic analysis, detailing their host, country of origin, GenBank accession numbers, and references.

Species	Isolate	Host	Country	GenBank Accession Number			References
				ITS	*TUB2*	*TEF1-α*	
*Biscogniauxia mediterranea*	Bm04.001	*Quercus suber* L.	Portugal	KM216752	KM267202	KM216788	[68]
*B. mediterranea*	Bm10.019	*Q. suber*	Portugal	KM216761	KM267210	KM216797	[68]
*Botryosphaeria corticis* (Demaree & Wilcox) Arx & E. Mull.	ATCC 22927	*Vaccinium corymbosum* L.	USA	DQ299247	EU673108	EU673291	[33,69]
*Bo. corticis*	CBS119047	*V. corymbosum*	USA	DQ299245	EU673107	EU017539	[33,69]
*Bo. corticis*	CBS119048	*V. corymbosum*	USA	DQ299246	MT592464	EU017540	[33,69]
*Bo. dothidea* (Moug. ex Fr.) Ces. & De Not.	CBS110302	*Vitis vinifera* L.	Portugal	AY259092	EU673106	AY573218	[70,71,72]
*Bo. dothidea*	CMW8000	*Prunus* sp.	Switzerland	AY236949	AY236927	AY236898	[63]
*Bo. dothidea*	BJS-01s	*V. vinifera*	China	JX275778	JX462259	JX462285	[49]
*Bo. pseudoramosa* G.Q. Li & S.F. Chen	CERC 2001	*Eucalyptus urophylla* × *Eucalyptus grandis*	China	KX277989	KX278198	KX278094	[73]
*Bo. pseudoramosa*	CERC 3455	*E. urophylla* × *E. grandis*	China	KX277997	KX278206	KX278102	[73]
*Bo. pseudoramosa*	CERC 3472	*E. urophylla* × *E. grandis*	China	KX277999	KX278208	KX278104	[73]
*Bo. qingyuanensis* G.Q. Li & S.F. Chen	CERC 2947	*E. urophylla* × *E. grandis*	China	KX278001	KX278210	KX278106	[73]
*Bo. qingyuanensis*	CERC 2946	*E. urophylla* × *E. grandis*	China	KX278000	KX278209	KX278105	[73]
*Bo. qingyuanensis*	TKBQ16-30	*Carya cathayensis* Sarg.	China	MW561677	MW561777	MW561687	[74]
*Diplodia bulgarica* A.J.L. Phillips, J. Lopes & S.G. Bobev	UCD11351	*Malus domestica* (Suckow) Borkh.	USA	OR631210	OR637362	OR637364	[75]
*D. bulgarica*	UCD11350	*M. domestica*	USA	OR631209	OR637361	OR637363	[75]
*D. bulgarica*	CBS124136	*M. sylvestris* (L.) Mill.	Bulgaria	MH863355	MT592475	GQ923822	[76,77,78]
*D. corticola* A.J.L. Phillips, A. Alves & J. Luque	CBS 112546	*Q. ilex* L.	Spain	AY259090	EU673117	EU673310	[70,72]
*D. corticola*	CBS 112549	*Q. suber*	Portugal	AY259100	DQ458853	AY573227	[70,71]
*D. corticola*	UCD1260So	*V. vinifera*	California	GU799470	GU799464	GU799467	[79]
*D. cupressi* A.J.L. Phillips & A. Alves	CBS168.87	*Cupressus sempervirens* L.	Israel	DQ458893	DQ458861	DQ458878	[80]
*D. cupressi*	CBS261.85	*C. sempervirens*	Israel	DQ458894	DQ458862	DQ458879	[80]
*D. cupressi*	CBS121027	*C. sempervirens*	Cyprus	MT587340	MT592487	MT592045	[78]
*D. mutila*	CBS112553	*V. vinifera*	Portugal	AY259093	MZ073931	AY573219	[70,71,81]
*D. mutila*	CBS230.30	*Phoenix dactylifera* L.	USA	DQ458886	DQ458849	DQ458869	[80]
*D. mutila*	PD75	Holly	USA	GU251119	GU251779	GU251251	[82]
*D. seriata* De Notaris	UCD244Ma	*V. vinifera*	California	DQ008314	DQ008337	EU012406	[79]
*D. seriata*	CBS 112555	*V. vinifera*	Portugal	AY259094	DQ458856	AY573220	[70]
*D. seriata*	SDZ-01	*V. vinifera*	China	HQ629954	HQ629956	HQ629958	[49]
*Dothiorella brevicollis* Jami, Gryzenh., Slippers & M.J. Wingf.	CMW36463	*Vachellia karroo* (Hayne) Banfi & Galasso	South Africa	JQ239403	JQ239371	JQ239390	[83]
*Do. brevicollis*	CMW36464	*V. karroo*	South Africa	JQ239404	JQ239372	JQ239391	[83]
*Do. brevicollis*	CBS130412	*V. karroo*	South Africa	MT587395	MT592578	MT592107	[78]
*Do. dulcispinae* Jami, Gryzenh., Slippers & M.J. Wingf.	CMW36460	*V. karroo*	South Africa	JQ239400	JQ239373	JQ239387	[83]
*Do. dulcispinae*	CMW36461	*V. karroo*	South Africa	JQ239401	JQ239374	JQ239388	[83]
*Do. dulcispinae*	CMW36462	*V. karroo*	South Africa	JQ239402	JQ239375	JQ239389	[83]
*Do. iberica* A.J.L. Phillips, J. Luque & A. Alves	CBS115041	*Q. ilex*	Spain	NR111165	EU673096	AY573222	[72,84]
*Do. iberica*	UCRDI3	*Prunus dulcis* (Mill.) D.A.Webb	California	KP012591	KP067201	KP828802	[85]
*Do. iberica*	UCRDI1	*P. dulcis*	California	KP012590	KP067200	KP828801	[85]
*Do. oblonga* F.J.J. van der Walt, Slippers & G.J. Marais	CBS130414	*V. karroo*	South Africa	MT587408	MT592598	MT592120	[78]
*Do. oblonga*	CBS121766	*Senegalia mellifera* (Vahl) L.A. Silva & J. Freitas	South Africa	EU101301	KX464863	EU101346	[86,87]
*Do. oblonga*	CBS121765	*S. mellifera*	South Africa	KF766163	KX464862	EU101345	[86,87,88]
*Do. sarmentorum* (Fr.) A.J.L. Phillips, Alves & Luque	CMW39366	*Aesculus hippocastanum* L.	Serbia	KF575009	KF575105	KF575047	[89]
*Do. sarmentorum*	PD78	*P. dulcis*	California	GU251169	GU251829	GU251301	[82]
*Do. sarmentorum*	CBS115038	*Malus pumila* Mill.	Netherlands	AY573206	EU673101	AY573223	[71]
*Lasiodiplodia crassispora* T.I. Burgess & P.A. Barber	UCD27Co	*V. vinifera*	California	GU799457	GU799480	GU799488	[90]
*L. crassispora*	CMW13488	*Eucalyptus urophylla* S.T. Blake	Venezuela	DQ103552	KU887507	DQ103559	[91,92]
*L. crassispora*	UCD24Co	*V. vinifera*	California	GU799456	GU799479	GU799487	[90]
*L. gonubiensis* Pavlic, Slippers & M.J. Wingf	CBS115812	*Syzygium cordatum* Hochst.	South Africa	DQ458892	DQ458860	DQ458877	[80]
*L. gonubiensis*	CMW36240	*Adansonia* sp.	Africa	KU887124	KU887502	KU887001	[92]
*L. gonubiensis*	CMW43763	*Bruguiera gymnorhiza* (L.) *Savigny*	South Africa	KU587955	KU587865	KU587944	[93]
*L. iranensis* Abdollahz., Zare & A.J.L. Phillips	PCoCo10	*Theobroma cacao* L.	Taiwan	OR534188	OR551954	OR552312	[94]
*L. iranensis*	PCoCo11	*T. cacao*	Taiwan	OR534189	OR551955	OR552313	[94]
*L. iranensis*	PCoCo12	*T. cacao*	Taiwan	OR534190	OR551956	OR552314	[94]
*L. pseudotheobromae* A.J.L. Phillips, A. Alves & Crous	CBS116459	*Gmelina arborea* Roxb. ex Sm.	Costa Rica	EF622077	EU673111	EF622057	[72,95]
*L. pseudotheobromae*	MFLUCC 18-1120	*Magnolia liliifera* (L.) L.	China	MK496933	MK524719	MK521585	[96]
*L. pseudotheobromae*	MFLUCC 18-0950	*M. liliifera*	China	MK501818	MK550605	MK521586	[96]
*L. theobromae* (Pat.) Griffon & Maubl.	CAA006	*V. vinifera*	USA	DQ458891	DQ458859	DQ458876	[80]
*L. theobromae*	GX-5-5A	*V. vinifera*	China	JX275780	JX462262	JX462288	[49]
*L. theobromae*	TJXHS1S1	*V. vinifera*	China	JX275790	JX462278	JX462304	[49]
*N. arbuti* (D.F. Farr & M. Elliott) Crous, Slippers & A.J.L. Phillips	CBS116576	*Arbutus menziesii* Pursh	USA	KX464156	KX464928	KX464651	[87]
*N. arbuti*	CBS116574	*A. menziesii*	USA	KX464154	KX464926	KX464649	[87]
*N. arbuti*	CBS116573	*A. menziesii*	USA	KX464153	KX464925	KX464648	[87]
*N. australe*	CMW6837	*Acacia* sp.	Australia	AY339262	AY339254	AY339270	[97]
*N. australe*	CMW9073	*Acacia* sp.	Australia	AY339261	AY339253	AY339269	[97]
*N. australe*	CMW6853	*Sequoiadendron giganteum* (Lindl.) J. Buchholz	Australia	AY339263	AY339255	AY339271	[97]
*N. brasiliense* M.W. Marques, A.J.L. Phillips & M.P.S. Camara	CMM1285	*Mangifera indica* L.	Brazil	JX513628	KC794030	JX513608	[98]
*N. brasiliense*	CMM1338	*M. indica*	Brazil	JX513630	KC794031	JX513610	[98]
*N. brasiliense*	CMM1269	*M. indica*	Brazil	JX513629	KC794032	JX513609	[98]
*N. hongkongense* G.Q. Li & S.F. Chen	CERC 2967	*Araucaria cunninghamii* Mudie	China	KX278050	KX278259	KX278155	[73]
*N. hongkongense*	CERC 2968	*A. cunninghamii*	China	KX278051	KX278260	KX278156	[73]
*N. hongkongense*	CERC 2973	*A. cunninghamii*	China	KX278052	KX278261	KX278157	[73]
*N. mangroviorum* J.A. Osorio, Jol. Roux & Z.W. de Beer	CPC32482	*Diospyros dichrophylla* (Gand.) De Winter	South Africa	MT587494	MT592701	MT592209	[78]
*N. mangroviorum*	CMW41365	*Avicennia marina* (Forssk.) Vierh.	South Africa	KP860859	KP860779	KP860702	[93]
*N. mangroviorum*	CMW42481	*Bruguiera gymnorhiza* (L.) Savigny,	South Africa	KP860848	KP860770	KP860692	[93]
*N. mangiferae* (Syd. & P.Syd.) Crous et al.	CMW7024	*Magnifera indica* L.	Australia	AY615185	AY615172	DQ093221	[99]
*N. mangiferae*	CMW7797	*M. indica*	Australia	AY615186	AY615173	DQ093220	[99]
*N. mangiferae*	CMW7081	*M. indica*	Australia	AY615187	AY615174	KF766425	[99]
*N. microconidium*	CERC 3497	*E. urophylla* × *E. grandis*	China	KX278053	KX278262	KX278158	[73]
*N. microconidium*	CERC 3498	*E. urophylla* × *E. grandis*	China	KX278054	KX278263	KX278159	[73]
*N. microconidium*	CBS118821	*Syzygium cordatum* Hochst.	South Africa	MT587497	MT592704	MT592212	[78]
*N. luteum* (Pennycook & Samuels) Crous, Slippers & A.J.L. Phillips	CBS110299	*V. vinifera*	Portugal	AY259091	DQ458848	AY573217	[70,71]
*N. luteum*	CBS110497	*V. vinifera*	Portugal	EU673311	EU673092	EU673277	[72]
*N. luteum*	CBS133502	*Persea americana* Mill.	USA	MT587483	MT592689	MT592197	[78]
*N. parvum* (Pennycook & Samuels) Crous, Slippers & A.J.L. Phillips	CBS110301	*V. vinifera*	Portugal	AY259098	EU673095	AY573221	[70,71,72]
*N. parvum*	CMW9081	*Populus nigra* L.	New Zealand	AY236943	AY236917	AY236888	[97]
*N. parvum*	CBS123652	*S. cordatum*	South Africa	KX464184	KX464996	KX464710	[87]
*N. parvum*	Zai-5	*Syzygium samarangense* Merr. & L.M. Perry	Taiwan	OR534045	OR551811	OR552360	[94]

## Data Availability

All sequence data are available in NCBI GenBank in accordance with the accession numbers in the manuscript.
